# Single-atom Cu anchored catalysts for photocatalytic renewable H_2_ production with a quantum efficiency of 56%

**DOI:** 10.1038/s41467-021-27698-3

**Published:** 2022-01-10

**Authors:** Yumin Zhang, Jianhong Zhao, Hui Wang, Bin Xiao, Wen Zhang, Xinbo Zhao, Tianping Lv, Madasamy Thangamuthu, Jin Zhang, Yan Guo, Jiani Ma, Lina Lin, Junwang Tang, Rong Huang, Qingju Liu

**Affiliations:** 1 Yunnan Key Laboratory for Micro/Nano Materials & Technology, National Center for International Research on Photoelectric and Energy Materials, School of Materials and Energy, Yunnan University, Kunming, 650091 China; 2 Department of Chemical Engineering, University College London, London, WC1E 7JE UK; 3 Key Laboratory of Synthetic and Natural Functional Molecule of the Ministry of Education, the energy and Catalysis Hub, College of Chemistry and Materials Science, Northwest University, Xi’an, 710127 China; 4 Key Laboratory of Polar Materials and Devices (MOE) and Department of Electronics, East China Normal University, Shanghai, 200062 China

**Keywords:** Photocatalysis, Hydrogen energy, Photocatalysis

## Abstract

Single-atom catalysts anchoring offers a desirable pathway for efficiency maximization and cost-saving for photocatalytic hydrogen evolution. However, the single-atoms loading amount is always within 0.5% in most of the reported due to the agglomeration at higher loading concentrations. In this work, the highly dispersed and large loading amount (>1 wt%) of copper single-atoms were achieved on TiO_2_, exhibiting the H_2_ evolution rate of 101.7 mmol g^−1^ h^−1^ under simulated solar light irradiation, which is higher than other photocatalysts reported, in addition to the excellent stability as proved after storing 380 days. More importantly, it exhibits an apparent quantum efficiency of 56% at 365 nm, a significant breakthrough in this field. The highly dispersed and large amount of Cu single-atoms incorporation on TiO_2_ enables the efficient electron transfer via Cu^2+^-Cu^+^ process. The present approach paves the way to design advanced materials for remarkable photocatalytic activity and durability.

## Abstract (web summary)

In this work, the highly dispersed and large loading amount (>1 wt%) of copper single-atoms were achieved on TiO_2_, resulting into an apparent quantum efficiency of 56% at 365 nm, in addition to an excellent thermal stability as proved after storing 380 days.

## Introduction

Hydrogen evolution from solar-driven water splitting is a promising alternative strategy for future energy convertion^1–4^. Titanium oxide (TiO_2_) has been vastly employed^5^ and is still used as a benchmark photocatalyst for water splitting^6^ as it is low cost and more importantly, extremely stable and high efficiency under UV. Even so, TiO_2_ still suffers from high charge-carrier recombination and sluggish proton reduction kinetics^7^. Hence, addressing these issues is essential to enhance the charge separation and to promote H_2_ fuel synthesis. Several strategies such as doping, defect engineering, heterojunction formation, morphology variation, etc., have been reported to reduce the charge-carrier recombination and improve the H_2_ evolution^8^. Amongst them, loading a cocatalyst on the surface of the TiO_2_ is proved as an appropriate approach to enhance the charge separation through the established metal-semiconductor Schottky junction, which not only extracts the photogenerated electrons but also dramatically reduces the energy barrier for proton reduction.

Noble metals such as Pt, Au, and Pd are commonly used as cocatalysts in photocatalysis due to their low activation energy and efficient charge separation. For example, Pt loading improved TiO_2_ for H_2_ production by a factor of 12^9^. However, they are not only rare elements and hence high cost but also the efficiency achieved is still moderate. Recently, there were many studies on earth-abundant transition metals (e.g., Cu, Ni, Co, and Fe) to substitute these noble metals as a suitable alternative for photocatalysis^10–13^. On the other hand, all these cocatalyst-loaded photocatalysts still struggle to achieve a breakthrough inefficiency due to the low-atom utilization while the cocatalysts are in their bulk composition. Very recently, single-atom catalysts (SACs) have been highly focused due to maximizing the reaction active sites, resembling the homogeneous catalysis^14,15^. The isolated and active metal atoms anchored onto the photocatalysts offer more water-molecule adsorption and active sites. So far, SACs loaded TiO_2_ have been investigated for H_2_ evolution^6,16^, CO_2_ reduction^17^, and dye degradation^18^. However, the aggregation of SACs is inevitable during the catalytic reaction due to their high surface energy or leaching due to the unstable anchoring as the majority were synthesized by post-treatment (e.g., impregnation approach)^19–21^. More importantly, the larger the percentage of SACs, the higher the activity, whereas to load higher than 0.5 wt% of SACs is very challenging as the majority of the studies reported a limited amount of SACs (usually near 0.1–0.3 wt %)^1,2^ onto the high surface area of substrates and it is difficult to control and reproduce^4,22,23^. Hence, obtaining the highly dispersed and high concentration of SACs remains to be the main bottleneck in photocatalytic H_2_ production.

The easily-changed valence states of Cu nanoparticles have been a promising candidate for efficient charge separation and transfer, leading to higher catalytic performance compared to even noble metal loaded TiO_2_ samples^24–27^. A recent wrap-bake-peel process, using SiO_2_@M/TiO_2_@SiO_2_ as the intermediate following NaOH etching to produce Cu SACs, has achieved a benchmark apparent quantum efficiency (AQE) of 45.5% at 340 nm^6^. It is due to the reduction and regeneration of the active sites during the catalytic cycle. Such a success stimulates us to investigate a more efficient strategy to stabilize Cu SACs and more importantly to generate an in-situ self-heal approach for continuous H_2_ production from water, thus no need for the regeneration step. To achieve this, the intact interaction between the single atoms and the support is crucial to obtain atomically evenly-dispersed Cu^28,29^.

Here, we have developed a bottom-up approach, which is different from the post-treatment approach reported in the literature including the very recent report to obtain evenly dispersed SACs on the substrate^6,30^. The metal-organic framework (MOF) MIL-125 was first synthesized using it as a precursor. Then metal ions were anchored into the MOF MIL-125 to generate a metal-oxygen-titanium bond, which is the key to ensuring uniformly immobilized metal SAC on the final catalysts^31–33^. Finally, the metal-MIL-125 intermediates were calcined to synthesize the final photocatalysts. This new strategy ensures atomic dispersion of metal cocatalyst and enables to achieve a higher loading amount ~1.5 wt%. The optimised sample shows the photocatalytic H_2_ evolution rate of 101.7 mmol g^−1^ h^−1^ (or 2.03 mmol h^−1^). To make a straightforward comparison with the reported H_2_ evolution rate, we converted it to the widely used unit of mmol per unit mass per unit time under simulated solar light irradiation, somewhat higher than the best photocatalyst PtSA-TiO_2_ (95.3 mmol g^−1^ h^−1^). The CuSA-TiO_2_ exhibits AQE of 56% at 365 nm irradiation, exceeding all the state-of-the-art TiO_2_-based photocatalysts (AQE of 4.3–45.5%^6,34,35^).

## Results

### Photocatalytic properties of CuSA-TiO_2_

First, four sets of experiments were performed for AQE optimisation and the detailed conditions were presented in Supplementary Fig. 1. The AQE results of CuSA-TiO_2_ under different wavelength light irradiation (365 nm, 385 nm, 420 nm, and 520 nm) are shown in Supplementary Fig. 1a. It indicates a decrease with increasing the wavelength followed by a slight increase at 520 nm due to the Cu-induced defects absorption (Supplementary Fig. 14). Also, the AQE measurement using different amounts of the photocatalyst was carried out, indicating the CuSA-TiO_2_ mass can affect AQE (Supplementary Fig. 1b). The changing trend is likely due to the fact that the higher photocatalyst mass would scatter more light when it is over 50 mg. With the 2:1 methanol:water ratio, the AQE at different light intensities was also tested (Supplementary Fig. 1d). The AQE result collected from various ratios of methanol: water indicates that methanol facilitates the H_2_ evolution from water. When the light intensity increases from 200 W/m2 to 500 W/m2, the AQE shows a slight increase. Hence, The CuSA-TiO_2_ represents an efficient and low-cost photocatalyst for continuous renewable H_2_ production.

**Fig. 1:**
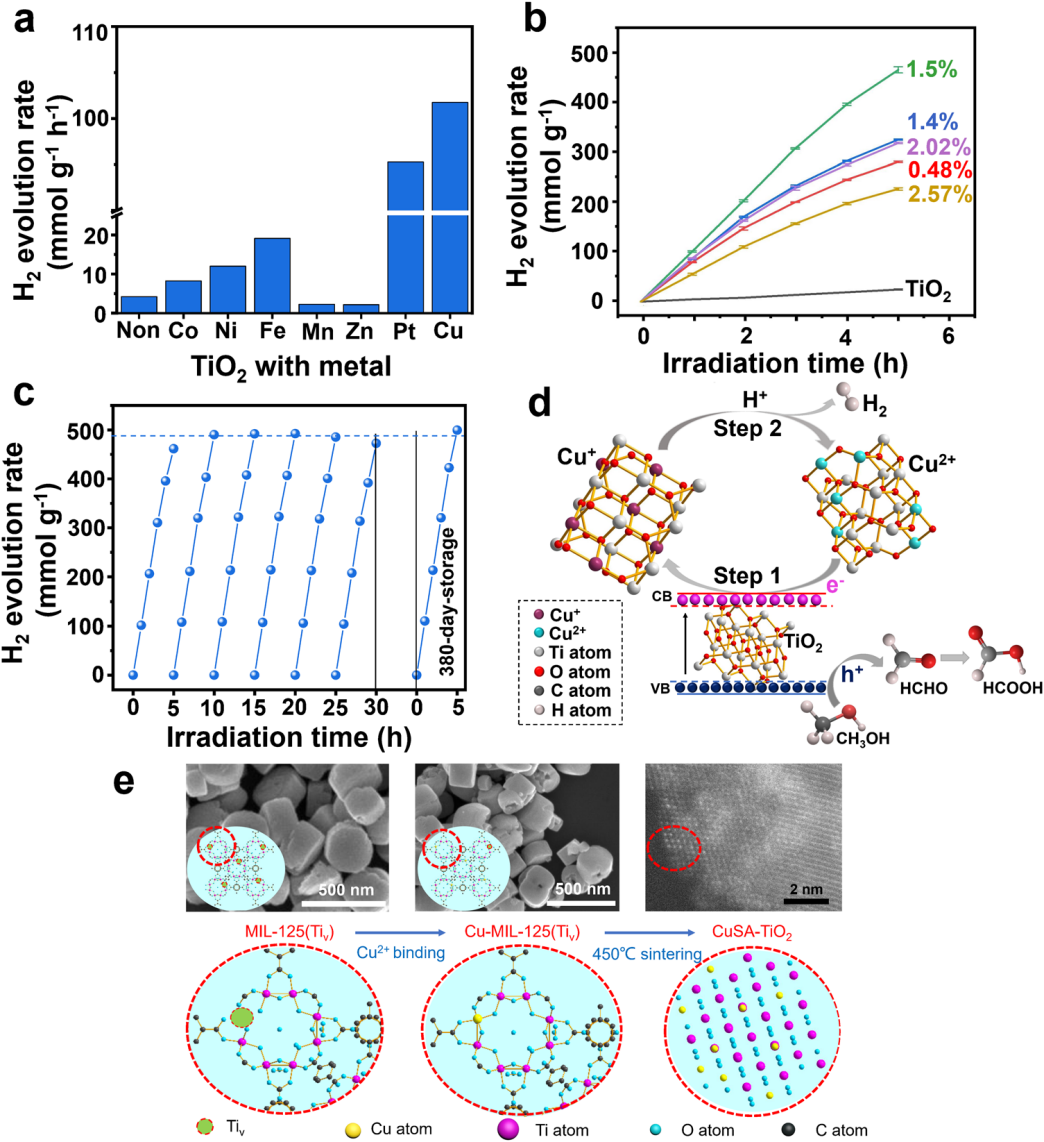
Photocatalytic H_2_ evolution performance and the formation of Cu SAC in the Ti lattice of TiO_2_. The photocatalytic H_2_ evolution rate of (**a**) TiO_2_ (non) and M-TiO_2_ derived from M-MiL-125(Ti_v_). **b** TiO_2_ and TiO_2_ loaded with different ratios of Cu SACs. **c** The photocatalytic activity of the ca. 1.5 wt% CuSA-TiO_2_ for six cyclic water splitting experiments and the last run is the activity of the sample after storing in the lab for 380 days. **d** The photocatalytic H_2_ evolution mechanism on 1.5 wt% CuSA-TiO_2_. **e** The corresponding schematic representation of the formation of copper SAC in the Ti lattice of TiO_2_, together with the related images.

First, the reference MIL-125 derived TiO_2_ was tested for photocatalytic H_2_ evolution under Xe lamp using methanol as a hole-scavenger. Fig. 1a shows the H_2_ evolution activities of TiO_2_ and other photocatalysts loaded with different metals such as Co, Ni, Fe, Mn, Zn, and Pt (0.75 wt% metal to precursor MIL-125(Ti_v_) before sintering). The samples were further analysed to determine the real amount of metal on TiO_2_ by an inductively coupled plasma test (Supplementary Table 1). All of them produce H_2_ higher than that of pristine TiO_2_ (4.2 mmol g^−1^ h^−1^) except Zn and Mn-TiO_2_, revealing that the metal single-atoms introduction plays a crucial role in the H_2_ evolution reaction. The activity order is CuSA-TiO_2_ (101.7 mmol g^−1^ h^−1^) > PtSA-TiO_2_ (95.3 mmol g^−1^ h^−1^) > FeSA-TiO_2_ (19.1 mmol g^−1^ h^−1^) > NiSA-TiO_2_ (12.0 mmol g^−1^ h^−1^) > CoSA-TiO_2_ (8.2 mmol g^−1^ h^−1^) > TiO_2_ (4.2 mmol g^−1^ h^−1^) > MnSA-TiO_2_ (2.3 mmol g^−1^ h^−1^) > ZnSA-TiO_2_ (2.2 mmol g^−1^ h^−1^). Interestingly, the CuSA-TiO_2_ evolve higher H_2_ than the benchmark Pt-loaded TiO_2_. The higher H_2_ evolution rate observed for the CuSA-TiO_2_ is due to the highly dispersed Cu SACs as proved later and their charge separation and catalytic effect as discussed below. Moreover, the larger loading amount of Cu SACs (1.5 wt%) compared with Pt SACs (0.64%) allows maximum utilization of the active sites to realize such an amazing activity, which might be ascribed to the easier coordination of dissociated Cu^2+^ with oxygen compared with [PtCl_4_]^2−^. Furthermore, the weight percentage of the Cu attached to the TiO_2_ was optimized as shown in Fig. 1b, and the highest H_2_ evolution activity is obtained on ca. 1.5 wt% CuSA-TiO_2_ (Supplementary Table 1). More importantly, an unprecedented AQE of 56% at 365 nm has been achieved on the ca. 1.5 wt% CuSA-TiO_2_, a new record. These results indicate that both the large atomic weight percentage and interaction of Cu with TiO_2_ are crucial for the extraordinary H_2_ evolution.

While increasing the Cu atoms amount beyond ca. 1.5 wt%, the decreased photocatalytic activity was observed. It is perhaps due to the limited pores available for higher Cu precursor anchoring, leading to screening the irradiated light. To show the experimental evidence of excessive Cu accumulation, we measured the HRTEM for 2.57 wt% CuSA-TiO_2_ and 1.5 wt% CuSA-TiO_2_, compared with pristine TiO_2_. Supplementary Figure 10 shows the patterns of 1.5 wt% CuSA-TiO_2_, which is similar to the pristine TiO_2_. While clear patterns of Cu nanoparticles (about 2–5 nm) observed in 2.57 wt% CuSA-TiO_2_ (Supplementary Fig. 2) are marked with arrows and circles. Hence, the poor activity observed for the Cu atom amount beyond ca. 1.5 wt% might be ascribed to the Cu accumulation, which inhibits the incident light from reaching the photocatalyst surface.

The long-term stability and reproducibility of the H_2_ evolution using the optimised CuSA-TiO_2_ were analyzed by the six consecutive photocatalytic water-splitting experiments under simulated solar light irradiation (Fig. 1c). In addition, the long-term experiment i.e., 20 days were also carried out for the stability test and results are shown in Supplementary Fig. 3. The sample was kept in the solution for each cycle, overall lasting for 20 days. The sample exhibits similar activity. Moreover, the photocatalyst after the long-term test was characterized by the ICP-AES, listed in Supplementary Table 1. The Cu amount of CuSA-TiO_2_ after the long-term test was estimated to be 1.54 wt%, which is similar to that of the fresh sample. The long-term activity and ICP-AES result further confirm the long-term stability and reproducibility of CuSA-TiO_2_.

It can be seen that no noticeable decrease was observed in the H_2_ evolution rate, suggesting that the prepared sample is highly stable and the results are reproducible. Furthermore, the photocatalytic activity of the 1.5 wt% CuSA-TiO_2_ after 380-day-storage in the lab was tested and we found that it remains the same as of the freshly prepared sample (Fig. 1c). Supplementary Table 2 lists the very recent progress on TiO_2_-based photocatalysts for water splitting, indicating TiO_2_ is still a highly attractive photocatalyst. Compared with these reports, one can see that our CuSA-TiO_2_ sample is two times more active than the reported ca 1 wt% Pt atom-TiO_2_ and six times better than the recently reported Cu-TiO_2_ in terms of specific mass evolution rate. Another finding is that it presents a record AQE of 56%, which is also more stable (>30 h). Such enhancement is believed to be due to the unique Cu states on TiO_2_ prepared by our pre-encapsulation synthesis approach.

### Morphology and structure characterization of CuSA-TiO_2_

**Fig. 2:**
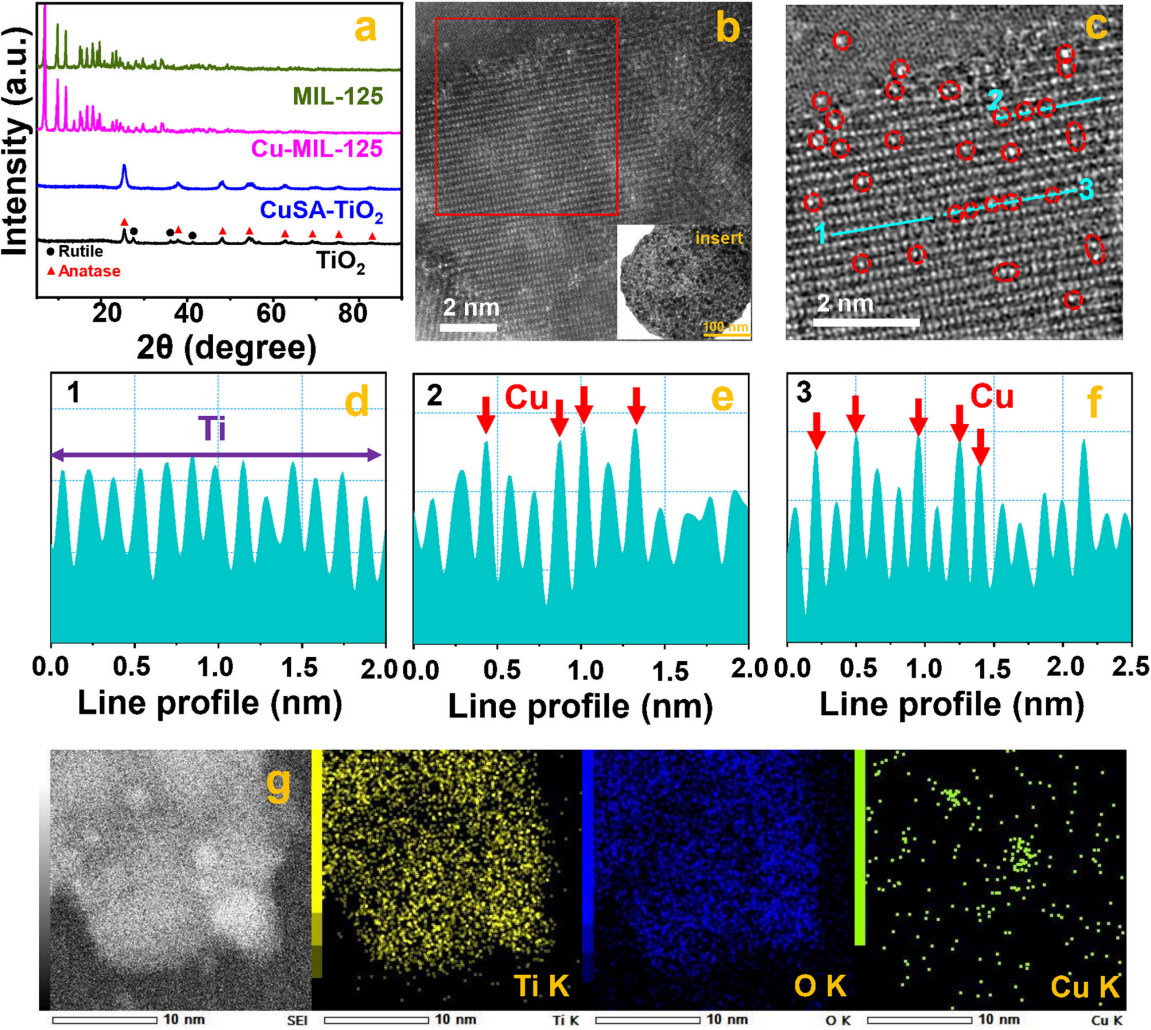
Structure and micromorphology of CuSA-TiO_2_. **a** The XRD images of MIL-125, Cu-MIL-125, TiO_2,_ and CuSA-TiO_2_. **b** HAADF STEM raw image of CuSA-TiO_2_. (Insert: low magnification TEM images of CuSA-TiO_2_). **c** Filtered HAADF STEM image from the area highlighted with a red rectangle in b and the corresponding line scan profiles. **d**–**f** Line 1, Line 2, and Line 3 marked in Fig. **c**. **g** STEM-EDS mapping of Ti, O, and Cu of the fresh CuSA-TiO_2_.

To explore the science behind the outstanding photocatalytic performance of 1.5 wt% CuSA-TiO_2_ (named CuSA-TiO_2_ subsequently), a series of studies were performed. Firstly, the X-ray diffraction (XRD) patterns of the as-prepared MIL-125(Ti_v_) were observed as shown in Fig. 2a, which agrees well with the earlier report^36^, suggesting that the MOF precursor was prepared successfully. The precursor MOF (MIL-125(Ti_v_)) was also observed with a regular cake-like morphology, the high specific surface area (SSA, 1361 m^2^ g^−1^, Supplementary Fig. 4a–b) and favorable pores^31^, which facilitates the Cu ions anchoring, leading to the formation of CuSA-TiO_2_ with the SSA of 294 m^2^ g^−1^ (Supplementary Fig. 4c–d). After incorporating the Cu in MIL-125(Ti_v_), the XRD pattern is nearly identical to that of the pristine MIL-125(Ti_v_), indicating that Cu^2+^ is encapsulated into the framework of MIL-125(Ti_v_) with high dispersity^38^. The same conclusion can be drawn from the sintered samples (TiO_2_ and CuSA-TiO_2_). Furthermore, the addition of Cu does not change the phase of TiO_2_^37^, so that there is only an anatase crystal structure existing in the CuSA-TiO_2_. Furthermore, the Zeta potential test of MIL-125(Ti_v_) indicates a potential value of −40.6 mV (Supplementary Fig. 5), that means the dispersion of MIL-125(Ti_v_) in water is stable with such large negative charges. More importantly, the positive Cu^2+^ ions can be readily electrostatically bonded to the Ti vacancies of MIL-125(Ti_v_) via M-O-Ti structure after adding the CuCl_2_ precursor aqueous solution. The Cu-encapsulated MIL-125(Ti_v_) (Cu-MIL-125(Ti_v_)) was then sintered at 450 °C to form Cu SACs on TiO_2_ (CuSA-TiO_2_). Such catalyst synthesis temperature was derived from the thermogravimetric differential thermal (TG-DTA) measurement (Supplementary Fig. 6). When the temperature is higher than 450 °C (black curve), the weight loss tends to be stable, which might be explained by the removal of organic ligands and stabilisation of Cu species at that temperature. Moreover, the actual ratio of Cu SACs on TiO_2_ is up to 1.5wt%, indicating the proposed strategy not only ensures the highly dispersed Cu anchoring but also achieves a rather large loading amount of Cu SACs.

Figure 1e shows the synthesis of the final catalyst from the intermediate MOF. The cake-shaped morphology of the intermediates (Supplementary Figure 7) was collapsed to form TiO_2_ or CuSA-TiO_2_ with clear lattice fringes, upon calcination of the MIL-125(Ti_v_), or Cu-MIL-125(Ti_v_). Supplementary Figs. 8 and 9 show the transmission electron microscopy (TEM) image of the MIL-125 derived TiO_2_ and the CuSA-TiO_2_, respectively. The collapsed morphology has significant merits as it can allow more Cu atoms exposure. The incorporation of Cu atoms on the Ti vacancy sites in the intermediate MOF is important as indicated in Fig. 1e, which not only stabilises Cu ions but also ensures single atom distribution of Cu in the prepared photocatalysts. This was investigated by the high-angle annular dark-field (HAADF) STEM (Fig. 2b–c, Supplementary Figs. 10–12). The bright contrast spots can be clearly seen only on the Ti atomic row. These bright spots are attributed to Cu as indicated in Supplementary Fig. 11f–i, confirming that Cu atoms are exclusively present in the Ti vacancies and other Cu configurations (e.g., clusters or nanoparticles) are not detected^8^. The line scan profiles marked with three blue lines randomly selected are shown in Fig. 2d–f, where lines 1 only contains Ti atoms while line 2 and 3 have both Ti and Cu atoms, confirming that there are Cu-O-Ti clusters.

The STEM-EDS mapping (Fig. 2g, Supplementary Fig. 12e) and EDS spectrum of CuSA-TiO_2_ (Supplementary Fig. 13) verify the existence of Ti, O, and Cu elements. Due to the porous nature and unsaturated bonds of MIL-125(Ti_v_), the Cu single atoms can be well stabilized. The stability of Cu atoms is further studied by measuring the HAADF-STEM of CuSA-TiO_2_ after 24-h photocatalytic reaction (Supplementary Figure 12). The line scan profile and STEM-EDS demonstrates that CuSA is still well dispersed on TiO_2_, confirming the excellent stability and strong anchoring of CuSA on TiO_2_, which is the key reason that our photocatalyst not only shows the record activity but also does not require regeneration as reported by others^6^.

### Photo-electric characterization of CuSA-TiO_2_

**Fig. 3:**
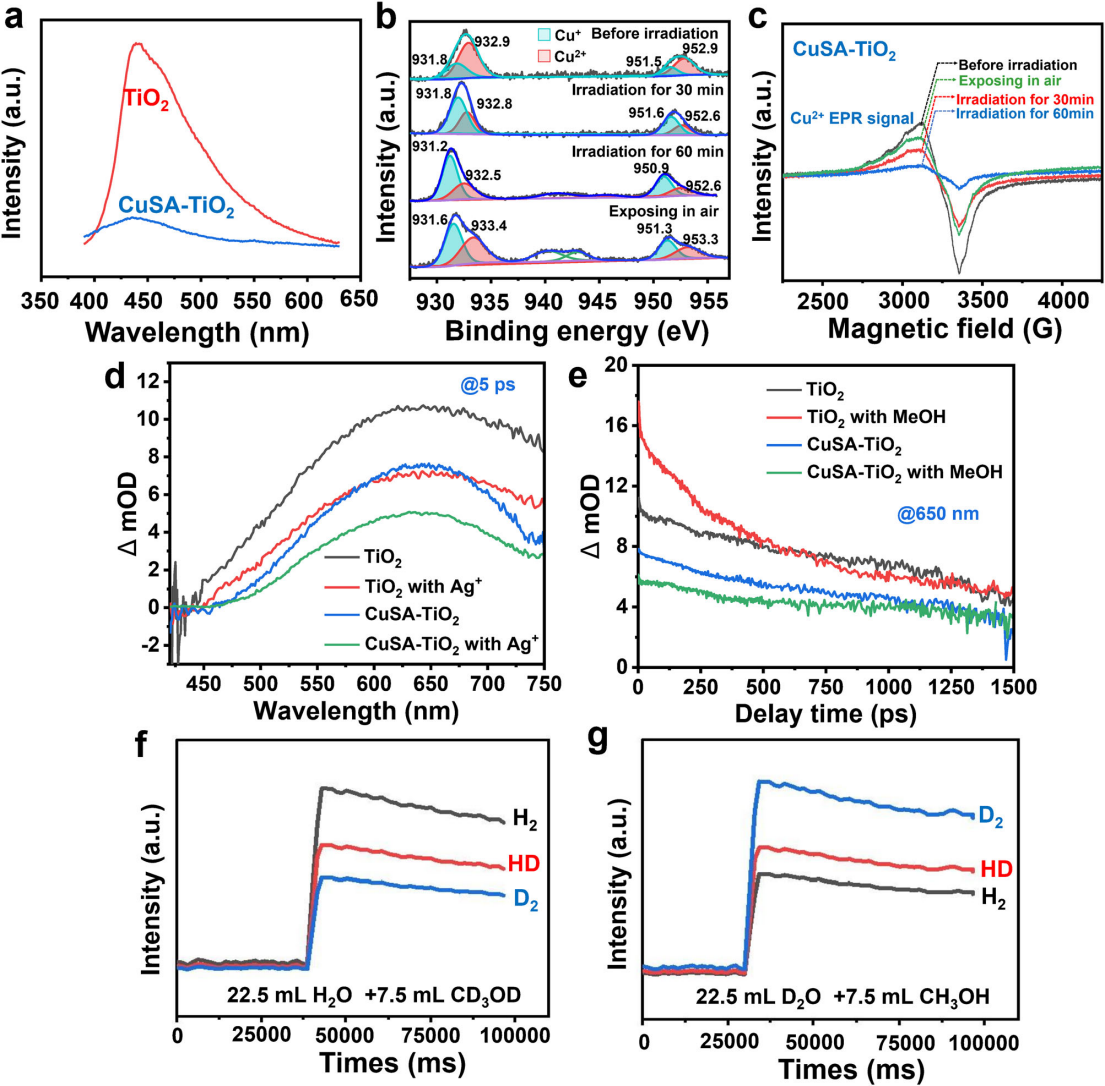
Carrier transfer mode and carrier dynamics of CuSA-TiO_2_. **a** Photoluminescence spectra of TiO_2_ and CuSA-TiO_2_ under an excitation wavelength of 375 nm. **b** In-situ Cu 2*p* XPS of CuSA-TiO_2_. **c** In-situ electron paramagnetic resonance spectra of CuSA-TiO_2_ with various states. **d** Transient absorption spectra of TiO_2_ and CuSA-TiO_2_ in the absence and presence of AgNO_3_ (2 mM), monitored in argon atmosphere after 320 nm excitation. **e** Charge dynamics decay in TiO_2_ and CuSA-TiO_2_ with and without methanol (10%) monitored at 650 nm. **f** The spectra of H_2_, HD, and D_2_ evolution on CuSA-TiO_2_ from the solution of CD_3_OD in H_2_O. **g** The spectra of H_2_, HD, and D_2_ evolution on CuSA-TiO_2_ from the solution of CH_3_OH in D_2_O.

UV-visible absorption spectroscopy (UV-vis) was used to explore the optical properties of TiO_2_ and CuSA-TiO_2_. The pristine TiO_2_ shows an absorption edge starting from 380 nm, corresponding to its wide bandgap transition (Supplementary Fig. 14). After loading CuSA, CuSA-TiO_2_ exhibits an obvious absorption in the visible region as well, a broad hump between 400 nm and 1050 nm, which is attributed to the d-d transition of Cu^2+^ state^6^. It is also evidenced from Supplementary Fig. 14 that the TiO_2_ absorption remains unchanged after loading CuSA. The transfer and separation efficiency of photogenerated charge carriers was studied by photoluminescence (PL) experiments. Figure 3a shows the PL spectra of TiO_2_ and CuSA-TiO_2_ excited at 375 nm. It is obvious that the pristine TiO_2_ shows high emission intensity due to the serious charge carrier recombination, which is significantly reduced after loading CuSA, indicating that the CuSA loading might effectively facilitate the photogenerated electrons extraction to the Cu active sites^39,40^. It is possible because the reduction potential of Cu^2+^/Cu^+^ (0.16 V vs. NHE) is more positive than the conduction band of TiO_2_ (−0.1 V vs. NHE)^41,42^.

PL lifetime is the average time that the fluorophore stays in the excited state before emission occurs^43^. The instrument response function (IRF) was first measured to be 530 ps (Supplementary Fig. 15). The PL decay tests for TiO_2_ and CuSA-TiO_2_ were then performed with 375 nm excitation beam taking into account TiO_2_ absorption spectra (Supplementary Fig. 15) and observed at 430 nm. The decay kinetics were fitted with a biexponential function as shown in Supplementary Table 3 after considering this IRF: *I*(t) = B_1_ exp(−t/τ_1_) + B_2_ exp(−t/τ_2_), where τ_1_ and τ_2_ are the decay times for the faster and slower components, respectively, B_1_ and B_2_ are the contributions of each component. The carrier lifetime of TiO_2_ and CuSA-TiO_2_ were determined to be 3.82 ns and 2.04 ns, respectively. The CuSA-TiO_2_ shows a shorter PL lifetime compared to that of TiO_2_, revealing the faster electron transfer in CuSA-TiO_2_^22,44^.

To further understand the charge carrier separation, photoelectrochemical experiments were performed and the photocurrents observed for TiO_2_ and CuSA-TiO_2_ are shown in Supplementary Fig. 16a. CuSA-TiO_2_ exhibits 3 times higher photocurrent than the pristine TiO_2_, indicating the more efficient carrier separation in CuSA-TiO_2_ following photoexcitation. This might be due to Cu-O as the bridge for more efficient carrier transfer to Cu active sites for H_2_ generation^45^. In addition, the photocurrent response of CuSA-TiO_2_ shows high reproducibility and stability for several on/off cycles. Moreover, the electrochemical impedance spectra (EIS) were used to study the transfer properties of charge carriers^46^, as indicated in Supplementary Fig. 16b. CuSA-TiO_2_ shows similar but dramatically decreased EIS Nyquist curves compared to pristine TiO_2_, indicating the CuSA species serve as electron acceptors, thus facilitating the interfacial charge separation. The efficient electron mobility is possibly ascribed to the reversible redox process between Cu^2+^ and Cu^+^ in CuSA-TiO_2_. Then, the electrochemical linear sweep voltammetry of CuSA-TiO_2_ was performed and shown in Supplementary Fig. 17, showing an overpotential of −0.72 V vs RHE for H_2_ production.

### Valence state characterization of Cu SAs

To examine the real chemical states of the photocatalysts during the photocatalytic reaction, in-situ x-ray photoelectron spectroscopy (XPS) spectra of CuSA-TiO_2_ before and during the reaction were monitored (Fig. 3b). The Cu 2*p* before irradiation appears to be composed of four Gaussian peaks, where 952.9 eV and 932.9 eV are assigned to the Cu 2*p*1/2 and Cu 2*p*3/2 of Cu^2+^ (70.58%, Supplementary Table 4), respectively. Another set of peaks at 951.5 eV and 931.8 eV are likely associated with Cu^+^ species^37^ (29.42%), illustrating the Cu atoms bonded with TiO_2_ rather than physical adsorption on the surface. After 30 min irradiation, the Cu^+^ content increases to 61.68% and remains at 62–66% after that till the end of the reaction. Interestingly, after exposing the used catalyst in the air for 10 min, the ratio of Cu^+^ decreases to 53.45%, and the satellite peak of Cu^2+^ appears, which is ascribed to the Cu^+^ is partially oxidised to Cu^2+^ again. Also, Ti 2*p* and O 1 *s* of CuSA-TiO_2_ and reference PtSA-TiO_2_ before and after 30 min irradiation were compared in Supplementary Fig. 18. For CuSA-TiO_2_, the content of surface Ti^3+^ increases from 0.00% to 47.02% as well as oxygen vacancy (Vo) increases from 8.81% to 18.21% due to light irradiation (Supplementary Table 5), that is believed due to the photogenerated electrons accumulation on both Ti^4+^ and Cu^2+^ species^47,48^. For PtSA-TiO_2_, only 13.12% Ti^3+^ was formed by irradiation (Supplementary Fig. 18d), which can be explained by the stronger carrier exchange between Pt and TiO_2_ compared to Cu and TiO_2_. The Pt 4 *f* spectrum of PtSA-TiO_2_ shows two peaks located at 74.4 eV and 70.9 eV, corresponding to Pt^2+^. After irradiation, the new peak at 69.2 eV appears, corresponding to the metallic Pt, suggesting the partial reduction of Pt^2+^. The O 1 *s* spectrum of PtSA-TiO_2_ (Supplementary Fig. 18f) shows the peak at 530.8 eV before irradiation assigning to the trace oxidation of Pt, which vanishes after irradiation, and Vo increases from 11.20% to 22.00% (Supplementary Table 6), matching well with that of Pt 4*f*. Interestingly, although Vo change is very similar for both CuSA-TiO_2_ and reference PtSA-TiO_2_, the Ti^3+^ in the former is much higher than the latter, indicating more photoelectrons transfer from the excited TiO_2_ to Pt^2+^ than that to Cu^2+^, which is reasonable as the reduction potential of Pt^2+^/Pt^0^ (0.758 eV) is much more positive than that of Cu^2+^/Cu^+^ (0.153 eV)^49^. We think there is little Cu metal on the surface of TiO_2_ due to the strong interaction between Cu and substrate as mentioned above and one-electron reduction process (Cu^2+^ to Cu^+^) is easier than two-electron process (Cu^2+^ to Cu^0^), which is crucial for the Cu^2+^ and Cu^+^ cycle (or self-healing) as indicated in Fig. 1d. The valence states of Cu in CuSA-TiO_2_ before and after irradiation were further confirmed by in-situ electron paramagnetic resonance (EPR) (Fig. 3c). Before irradiation, the EPR spectrum of CuSA-TiO_2_ shows a strong signal of Cu^2+^, which decreases after 30 min irradiation and then increases after exposure to the air. This result indicates that EPR-silent Cu^+^ was formed during irradiation and then oxidized back to Cu^2+^ when exposed to air.

### Charge dynamics analysis

To further confirm the charge dynamics of the excited TiO_2_ and CuSA-TiO_2_, the femto second transient absorption spectra (fs-TAS) were monitored and shown in Supplementary Fig. 19 and Fig. 3. Figure 3d shows the TAS-signals of TiO_2_ and CuSA-TiO_2_ with and without AgNO_3_ (2 mM) under 320 nm excitation. For TiO_2_, the monotonically strong background is ascribed to the excited photoelectrons either in the conduction band or the trap states^50^. After introducing Ag^+^ as an electron scavenger, the TAS signal of TiO_2_ exhibits the decreased signal intensity, indicating this wavelength range is the fingerprint of photoelectrons. When adding Cu SAC onto TiO_2_, the TAS is nearly identical to that of TiO_2_ in the presence of Ag^+^ ions, clearly indicating that photogenerated electrons are successfully captured by the Cu^2+^ or the efficiency of Cu^2+^ to abstract photoelectrons from TiO_2_ is close to that of Ag^+^ ions, which is believed due to the strong chemical bond interaction in the Cu-O-Ti clusters. For CuSA-TiO_2_ in the presence of Ag^+^, the TAS signal further decreases due to the electron scavenging by both Cu^2+^ and Ag^+^. On the other hand, when adding methanol as a hole scavenger to TiO_2_, the enhanced photoelectron signal is observed at long wavelengths compared with that in the absence of methanol (monitored at 650 nm in Fig. 3e) due to the hole scavenging by methanol. This photoelectron dynamics decay is likely due to the reaction of oxidized species of methanol with the electrons. As expected, CuSA-TiO_2_ shows similar features of photoelectron decay in the presence or absence of methanol, confirming that electrons can be efficiently trapped by Cu^2+^ when holes are scavenged by methanol. The TAS results are well consistent with in-situ XPS and in-situ EPR results.

### Isotopic experiments

To differentiate the origin of the evolved H_2_, isotopic tracing experiments were performed on CuSA-TiO_2_. Figure 3f and Supplementary Fig. 20 exhibit the spectra of generated H_2_ from deuterated methanol and water (CD_3_OD/H_2_O). H_2_ is the major product, then HD, and finally deuterium (D_2_). This indicates that the source of protons is mainly derived from water and partially from methanol, consistent with others^51^. The same conclusion can be drawn from the other case study using deuterated water and methanol (D_2_O/CH_3_OH), as shown in Fig. 3g and Supplementary Fig. 21. Again, D_2_ shows the largest signal, then HD and finally H_2_, proving water is the major hydrogen source for H_2_ evolution.

### Theoretic calculations of carrier transfer

**Fig. 4:**
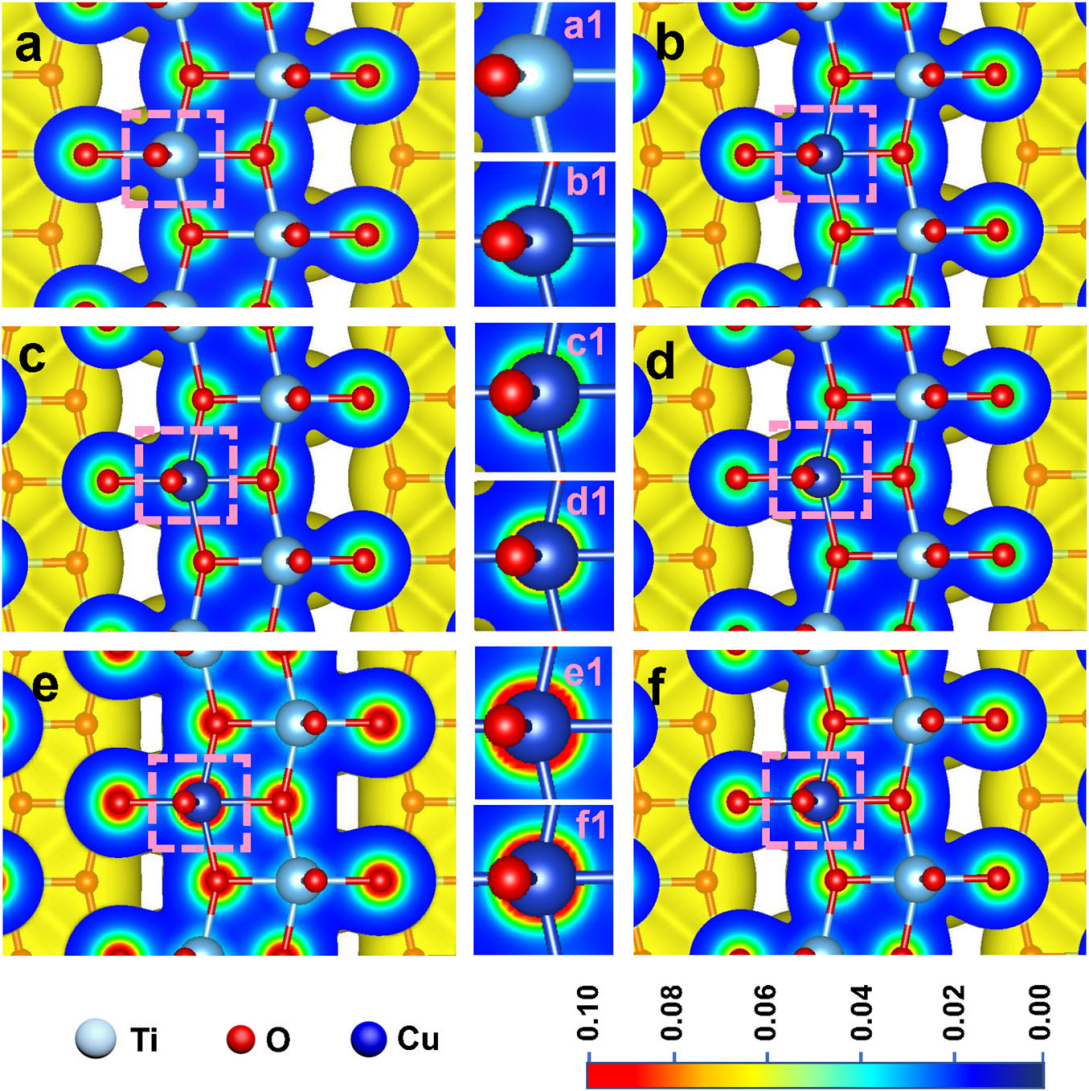
Charge density distribution with or without irradiation. **a** Charge density distribution of pure TiO_2_. **b** CuSA-TiO_2_ under dark condition (Cu is in the dotted square). **c** CuSA-TiO_2_ after 100 ps irradiation. **d** CuSA-TiO_2_ after 200 ps irradiation. **e** CuSA-TiO_2_ after 1 ns irradiation. **f** CuSA-TiO_2_ after turning off the irradiation for 100 ps. In the middle panel, (**a**1–**f**1) is the magnified Cu atom highlighted by pink square in (**a**–**f**). Black and red colored circles indicate more electrons’ accumulation on Cu.

The photogenerated electrons transfer to the CuSA and their dissipation was modelled by the DFT simulation. The CuSA-TiO_2_ sample was modelled using 1.5 wt% of Cu replacing Ti (Supplementary Fig. 22). The charge density diagrams of pristine TiO_2_ and CuSA-TiO_2_ under dark conditions are shown in Fig. 4a, b, respectively. It can be seen that when Ti is replaced by Cu (highlighted with a pink dotted square), the unbalanced charges lead to a slight accumulation of electrons on Cu. The gradual increase of electron density around Cu is obvious after irradiation for 100 ps (Fig. 4c), 200 ps (Fig. 4d), and 1 ns (Fig. 4e). While the irradiation stops, the charge density on Cu after 100 ps is obviously reduced (Fig. 4 f), indicating that the accumulated electrons reduce the Cu^2+^ to Cu^+^. At the same time, the Cu^+^ can be oxidized back to Cu^2+^ when no more photogenerated electrons are transferred to Cu while in contact with oxygen.

The DFT result for the pristine TiO_2_ after irradiation as shown in Supplementary Fig. 23. It can be seen that there is no increase of the charge density on Ti atoms until 1 ns (d1), which is back to the original state when irradiation stops. It suggests the lower charge density and slower charge mobility of pristine TiO_2_ compared with CuSA-TiO_2_. To make it clear, the selected areas in 4e and 4 f of Fig. 4 are magnified and compared in Supplementary Figure 24. The black circles and corresponding distances D_1_ and D_2_ (Supplementary Fig. 24) are used to present the charge density on the Cu atom after irradiation for 1 ns and after turning off the irradiation for 100 ps. Obviously, the D_1_ is larger than D_2_, indicating that the charge density on the Cu atom after irradiation for 1 ns is quite high while the obviously reduced density is observed when stopping the irradiation after 100 ps.

The surface photovoltage (SPV) was measured by the Kelvin probe (KP) to monitor the charge separation on CuSA-TiO_2_ and PtSA-TiO_2_ (Supplementary Fig. 25a–b). The ΔSPV comparison before and after irradiation for CuSA-TiO_2_ and PtSA-TiO_2_ has been shown in Supplementary Figure 25c. The average ΔSPV before and after irradiation for CuSA-TiO_2_ is 185 mV, which is higher than that of PtSA-TiO_2_ (144 mV), indicating the more enhanced charge separation rate in CuSA-TiO_2_.

### Hydrogen evolution mechanism

The dramatic enhancement in photocatalytic H_2_ evolution activity of CuSA-TiO_2_ can be attributed to the following reasons. First of all, the synthesis strategy provides suitable sites (Ti vacancy) and a high specific surface area for Cu atoms stabilization (Fig. 1e). Secondly, the abundant Cu^2+^ effectively traps the photogenerated electrons, leading to the significantly reduced recombination of the photogenerated charges (step 1 in Fig. 1d). The Cu^+^ has a positive potential to reduce H_2_O to H_2_ (step 2 in Fig. 1d), then Cu^+^ returns to Cu^2+^. Such an interesting reversible process or in-situ self-healing enables CuSA-TiO_2_ to achieve higher photocatalytic activity than the conventional Pt/TiO_2_^51^. It is also higher than other single atom sites decorated photocatalysts reported due to such a high concentration of Cu (1.5 wt%) anchored on the surface of TiO_2_. The interaction between Cu^+^ and H_2_O is also verified by density functional theory (DFT) calculation. The H_2_O reduction by Cu^+^ needs to overcome the energy barrier of 0.44 eV and is exothermic by 0.83 eV (Supplementary Fig. 26). To verify the oxidized products of methanol, the solution of D_2_O/CH_3_OH after the reaction was tested by nuclear magnetic resonance (NMR) and detected the by-product HCOOH (Supplementary Fig. 27), confirming that methanol was first oxidised to HCHO and further to HCOOH (Fig. 1d).

In summary, a reproducible and low-cost pre-encapsulation strategy was developed for stabilizing metal single atoms on TiO_2_ and was further demonstrated for solar fuel H_2_ synthesis. The strongly anchored Cu single atoms trigger a reversible/self-healing and continuous photocatalytic process, which has been proved by both experimental and theoretical studies. The synthesised CuSA-TiO_2_ shows the higher H_2_ evolution rate with the benchmark apparent quantum efficiency of 56% at 365 nm. The obtained higher activity is due to the advantage of MOF structure with extremely large surface area as the intermediate, which maximises the exposed sites for CuSA immobilization on TiO_2_, reaching ~1.5 wt%. After calcination, the strongly bonded CuSA on TiO_2_ effectively separates photoelectrons, and then electrons cascade to reduce water to H_2_, along with methanol oxidation. Both the diverse spectroscopic and in-situ experiments as well as DFT results reveal the most efficient charge separation by CuSA than any other cocatalysts and prove the significance of the in-situ self-healing effect of Cu species during the photocatalytic reaction. The present atomic-level photocatalytic material design strategy indeed paves the way towards a competitive H_2_ production for commercial application.

## Methods

All commercially available chemicals, reagents, and solvents were used as received without further purification unless noted otherwise.

### Preparation of MOF MIL-125(Ti_v_) precursor

The precursor of MIL-125 (MIL stands for Material from Institut Lavoisier) was prepared by following the reported procedure^1^. In a typical process, 3 g of terephthalic acid (1,4-benzenedicarboxylic acid) was added to the 54 ml of N,N dimethylacetamide (DMF) and magnetically stirred for 10 min. Then, 6 mL of methanol was added into the above mixture followed by the addition of 1.2 mL (to create Ti vacancies, we use 1.2 mL instead of 1.56 mL from the literature) of Ti(OC_4_H_9_)_4_ under the stirring condition for 5 min. After that, the solution was transferred into a 100 mL Schlenk tube and kept at 130 °C for 20 h. After cooling down to room temperature, the precipitate was separated by centrifugation and washed by DMF and methanol consecutively. The free solvent attached to the precipitate was removed by vacuum drying. The final obtained sample is MIL-125.

### Synthesis of SAC-MIL and preparation of final photocatalysts

Typically, 0.5 g of MIL-125 particles were dispersed in 40 mL of DI water, then the metal-salt with different weight ratios (e.g., 6.8 mg CuCl_2_ for 0.75 wt% Cu) was added into the MIL-125 dispersion and stirred for 3 h for encapsulated precursor synthesis. The resulting materials were then centrifuged and washed with water, dried at 80 ^o^C. Finally, the MSA-TiO_2_ particles (M = Cu, Co, Ni, Fe, Mn, Zn, and Pt) were obtained by annealing at 450 ^o^C for 4 h in the air.

### Procedure of photocatalysis

The photocatalytic water-splitting experiments were performed on the full glass automatic on-line trace gas analysis system (Fig. 28a, Labsolar-6A, Perfect Light Ltd.) and Multichannel photochemical reactor (Fig. 28b, PCX-50C, Perfect Light Ltd.). With Labsolar-6A, a Xe lamp (Perfect light PLS-SXE300C) equipped with filters was used as the simulated solar spectral source. (The data in Fig. 1(a–c) was collected with the light intensity of 500 W/m^2^, and that in Supplementary Fig. 3 was collected with the slightly reduced light intensity of 325 W/m^2^ due to the safety issue for a very long-time experiment.) The as-prepared catalyst (20 mg) was uniformly dispersed in 120 mL of H_2_O/methanol aqueous solution by using a magnetic stirrer (containing H_2_O/methanol with a ratio of = 1:2). The system was vacuum-treated several times to remove the dissolved air, and the amount of produced H_2_ was measured by an on-line gas chromatograph (GC7900). During the reaction, the temperature was maintained at 40 °C using water circulation. For cyclic experiments, the sample was collected through centrifugation and drying after the photocatalytic reaction, without any other treatment. The sample storage for 380 days was stored in a glass bottle in a normal lab cabinet.

The PCX-50C, a 1 W UV LED (Wavelength range: 365 nm, light intensity: 34.5 mW/cm^2^) was used as the simulated light source for AQE calculation. The as-prepared catalyst (50 mg) was uniformly dispersed in 30 mL aqueous solution under magnetic stirring (containing H_2_O/methanol, v/v = 1:2). The rest of the conditions were similar as stated above.

The AQE is calculated by using the following equation and the photocatalytic H_2_ evolution (PHE) rate obtained from PCX-50C (Supplementary Fig.28b):

1 $${{{{{\rm{AQE}}}}}}=\frac{2{{{{{{\rm{MN}}}}}}}_{{{{{{\rm{A}}}}}}}{{{{{\rm{hc}}}}}}}{{{{{{\rm{AIt}}}}}}\lambda }\times 100 \%$$

where M is the molar amount of hydrogen, N_A_ is the Avogadro’s constant, h is the Planck constant, c is the light velocity, I is the intensity of the light, A is the irradiation area measured by the reactor window with a diameter of 3 cm, t is the reaction time, and λ is the wavelength of light (365 nm)^1^.

The amount of hydrogen via 50 mg photocatalyst in the reactor was measured to be 0.753 mmol within 1 h at 40 °C.

### Characterization

The phase structures of the prepared samples were determined by the X-Ray diffraction (XRD) measurements using an X-ray diffractometer (Rigaku, Japan) with CuKa irradiation. The accelerating voltage and applied current were 40 kV and 80 mA, respectively. The morphology and microstructure of the samples were examined by emission scanning electron microscope (FE-SEM, Nova nanoSEM 450) and transmission electron microscope (TEM, JEM-2100). The high-angle annular dark-field (HAADF) STEM for CuSA-TiO_2_ was obtained using JEM-ARM300F equipment. The Brunauer–Emmett–Teller (BET) specific surface area of the prepared powders was analyzed by a 3H-2000PS2 sorption analyzer, and the porosity of the samples was evaluated based on nitrogen adsorption isotherms at 77 K. UV-vis diffused reflectance spectra of the samples were obtained using a Metash UV-9000S spectrophotometer. X-ray photoelectron spectroscopy (XPS) measurements were accomplished via a photoelectron spectrometer (Thermo ESCALAB 250Xi) with an Al Kα radiation source. The excitation wavelength was 320 nm, the scanning speed was 1200 nm min^−1^, and the PMT voltage was 700 V. The widths of the excitation slit and emission slit were both 5.0 nm. The Fourier transform infrared spectroscopy (FT-IR) spectra of starting materials and the as-synthesized samples were obtained using IR2000 equipment. Surface photovoltage (SPV) experiments were performed using the SKP5050 Kelvin probe. Electron spin resonance (EPR) spectroscopy was performed on Bruker EMXnano to detect the unpaired electrons of Cu^2+^ in CuSA-TiO_2_ powder at room temperature. Nuclear magnetic resonance (NMR, DRX500) was adopted to identify the product in the solution after the photocatalytic reaction.

**Photoluminescence (PL)** spectra and time-resolved fluorescence decay spectroscopy were obtained by an FLS 1000 fluorescence spectrophotometer (UK), where the sample powder was placed on a copper support. When testing the steady-state PL, 375 nm was selected for excitation. For PL decay testing, 375 nm and 430 nm were respectively selected for excitation and detection.

### Isotopic experiment

The isotopic experiment was performed using a multichannel photochemical reactor under 365 nm LED irradiation. 10 mg of CuSA-TiO_2_ was added into the solution of 22.5 mL of water and 7.5 mL of methanol, and irradiated for 24 h. Two separate experiments were carried out under the same conditions except for the deuterated part (CD_3_OD/H_2_O or D_2_O/CH_3_OH). Finally, the products were identified by Mass spectrum (MS, Hiden HPR-40).

### Inductively coupled plasma optical emission spectrometer (ICP) test

The ICP test was performed by PlasmaQuant PQ9000. The sample was dissolved in 5 mL freshly-made nitrohydrochloric acid at room temperature for 30 min and then heated to 136  ^o^C for another 20 min. After cooling down to room temperature, the solid residue was filtered followed by dilution to 50 mL for the test.

### Transient absorption spectroscopy (TAS) measurement

The fs-TAS measurement was carried out using a commercial transient absorption spectrometer (Newport TAS pump-probe system) that includes a 1 kHz Solstice (Newport Corp.) Ti:sapphire regenerative amplifier outputting 800 nm, 100 fs pulses. This laser light was split into two parts to generate the pump and the probe pulses. The tunable pump pulse was generated in a TOPAS-Prime (Light Conversion Ltd.) optical parametric amplifier and used to excite the sample at 320 nm. Broadband probe light (420–780 nm) was generated by focusing the Solstice output in a 2 mm sapphire crystal. Both the pump and probe beam overlapped spatially in the sample and the time delay between the pump and probe pulse was scanned by controlling the stage. 1 mg mL^−1^ sample was dispersed in an aqueous solution and transferred to 1 mm path length cuvettes. Samples were measured after purging with argon.

### Density functional theory (DFT) study

All calculations are performed with the DMOL3 module of Materials Studio 5.0. The ultrasoft pseudopotential was used in the calculation because of its several advantages in efficiency and veracity in the reciprocal space. The electronic exchange-correlation energy is treated within the framework of the generalized gradient approximation (GGA) with Perdew-Burke-Ernzerhof (PBE). The plan-wave expansion is truncated by the cutoff energy of 450 eV. The Monkhorst–Park scheme K-point grid sampling is set as 3 × 3 × 9 for the model. Mulliken population analysis is used to analyze the average net charge. The Mulliken population is defined as the electronic charge assigned to the atoms and atomic orbitals. The convergence value is set as 0.01 nm for the maximum displacement tolerances, 0.01 eV Å−1 for the maximum force in the geometrical optimization. The convergence accuracy of SCF is 10^−6^ eV/atom. The crystal structures and atom coordinates were also optimized firstly under the principle of energy minimization to obtain the appropriate cell parameters with stable structures for the model. Based on this principle, the electronic structures and optical properties could be calculated. After geometrical optimization, the lattice parameters of the pure anatase TiO_2_ are given as follows: a = b = 3.79 Å, c = 9.51 Å. The supercell built as 8 × 8 × 1, thus the crystal parameters of the optimized supercell are a = b = 30.28 Å, c = 9.51 Å. The simulation of irradiation was realized by adding an electromagnetic field to simulate the UV irradiation. The charge density of the model was recorded in a certain time interval, i.e., 100 ps, 200 ps, and 1 ns.

### Photoelectrochemical measurements

The photoelectrodes were prepared on fluorine-doped tin oxide (FTO) glass slides, which were cleaned with ethanol, rinsed with DI water, and dried before use. A 10 mg of the prepared samples were added into 200 μL of ethanol and 800 μL water with 10 μL of Nafion (5wt.%) and then carefully ground for a little while to form a homogenous slurry. Subsequently, the obtained slurry was evenly distributed onto the conductive side of FTO glass. After drying in the air, the photoelectron chemical properties of the obtained electrodes were tested in a three-electrode system using a CHI-760E electrochemical workstation. The prepared electrode, Pt wire, and Ag/AgCl electrode were used as the working, counter, and reference electrodes, respectively. The 0.2 M Na_2_SO_4_ aqueous solution was used as an electrolyte, and the photoelectrodes were irradiated using a 150 W xenon lamp with a light density of 95 mW/cm^2^. The photocurrents of the electrodes were measured using the amperometric (I–t curves) technique under repeatedly interrupted light irradiation. Electrochemical impedance spectroscopy (EIS) measurements were performed at an applied voltage of 5 mV with a frequency in the range of 10^5^–0.1 Hz.

### Statistical analysis

All data were presented as means with standard deviations (SD).

## Supplementary information

Supplementary Information

## Data Availability

The data that support the findings of this study are available from the corresponding author upon reasonable request. Source data are provided with this paper.
