# Author Correction: Single-atom Cu anchored catalysts for photocatalytic renewable H_2_ production with a quantum efficiency of 56%

**DOI:** 10.1038/s41467-022-29799-z

**Published:** 2022-04-11

**Authors:** Yumin Zhang, Jianhong Zhao, Hui Wang, Bin Xiao, Wen Zhang, Xinbo Zhao, Tianping Lv, Madasamy Thangamuthu, Jin Zhang, Yan Guo, Jiani Ma, Lina Lin, Junwang Tang, Rong Huang, Qingju Liu

**Affiliations:** 1 Yunnan Key Laboratory for Micro/Nano Materials & Technology, National Center for International Research on Photoelectric and Energy Materials, School of Materials and Energy, Yunnan University, Kunming, 650091 China; 2 Department of Chemical Engineering, University College London, London, WC1E 7JE UK; 3 Key Laboratory of Synthetic and Natural Functional Molecule of the Ministry of Education, the energy and Catalysis Hub, College of Chemistry and Materials Science, Northwest University, Xi’an, 710127 China; 4 Key Laboratory of Polar Materials and Devices (MOE) and Department of Electronics, East China Normal University, Shanghai, 200062 China

**Keywords:** Photocatalysis, Hydrogen energy, Photocatalysis

Correction to: *Nature Communications* 10.1038/s41467-021-27698-3, published online 10 January 2022.

In Supplementary Fig. 28b in the Supplementary PDF for this article, the figure panel incorrectly read ‘345 mW/cm2’ but should have been ‘34.5 mW/cm2’.

In the caption of Supplementary Fig. 20 in the Supplementary PDF for this article, the term ‘isotropic analysis’ should have read ‘isotopic analysis’.

In the caption of Supplementary Fig. 21 in the Supplementary PDF for this article, the term ‘isotropic analysis’ should have read ‘isotopic analysis’.

In the caption of Supplementary Fig. 28b in the Supplementary PDF for this article, the term ‘isotropic test’ should have read ‘isotopic test’.

The HTML has been updated to include a corrected version of the Supplementary Information.

## Supplementary information

Updated Supplementary Information

