# Supplementary material for: Single-atom Cu anchored catalysts for photocatalytic renewable H2 production with a quantum efficiency of 56%
Source: Nat Commun. 2022 Jan 10;13:58. doi: 10.1038/s41467-021-27698-3 (PMC8748625; doi:10.1038/s41467-021-27698-3)
Supplement: Supplementary file 1 — Supplementary Information [file 41467_2021_27698_MOESM1_ESM.pdf]

Supplementary Information

Single-atom Cu anchored catalysts for photocatalytic renewable H_2 production with a quantum efficiency of 56%

Yumin Zhang[1], Jianhong Zhao[1], Hui Wang[2], Bin Xiao[1], Wen Zhang[3], Xinbo Zhao[1], Tianping Lv[1], Madasamy Thangamuthu[2], Jin Zhang[1], Yan Guo[3], Jiani Ma[3], Lina Lin[4], Junwang Tang[2*], Rong Huang[4*], Qingju Liu[1*]

[1]Yunnan Key Laboratory for Micro/Nano Materials & Technology, National Center for International Research on Photoelectric and Energy Materials, School of Materials and Energy, Yunnan University, Kunming 650091, China. [2]Department of Chemical Engineering, University College London, London, WC1E 7JE, UK. [3]Key Laboratory of Synthetic and Natural Functional Molecule of the Ministry of Education, the energy and Catalysis Hub, College of Chemistry and Materials Science, Northwest University, Xi'an 710127, P. R. China. [4]Key Laboratory of Polar Materials and Devices (MOE) and Department of Electronics, East China Normal University, Shanghai 200062, china. [5]These authors contributed equally: Yumin Zhang, Jianhong Zhao, Hui Wang, Bin Xiao. Email: junwang.tang@ucl.ac.uk; rhuang@ee.ecnu.edu.cn; qjliu@ynu.edu.cn

Contents

1. Supplementary Figures

2. Supplementary Tables

1. Supplementary Figures

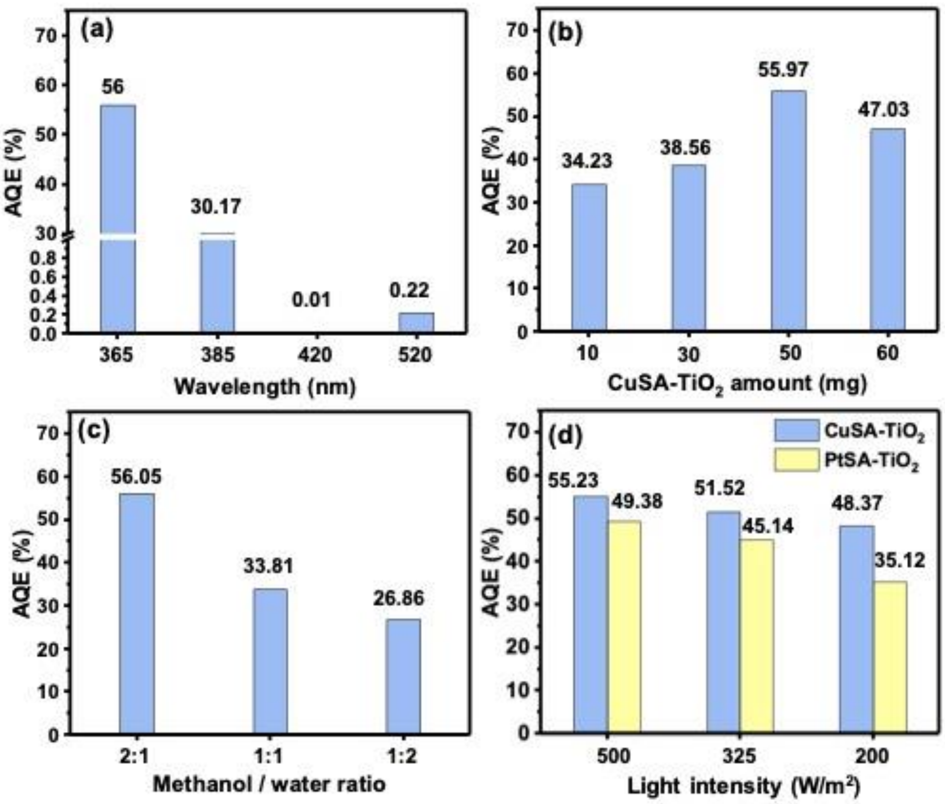

Supplementary Figure 1 | AQE test under different conditions. (**a**) AQE of CuSA-TiO_2 tested under the light sources with different wavelengths, light intensity: 500 W/m^2, catalyst amount: 50 mg, temperature: 40℃; The AQE dependency of wavelength has similar trend with the UV-vis adsorption of CuSA-TiO_2, showing a slight adsorption at 520 nm. (**b**) AQE of CuSA-TiO_2 tested with different catalyst amount, light source: 365 nm with the light intensity of 500 W/m^2, methanol to water ratio: 2:1, temperature: 40℃; (**c**) AQE of CuSA-TiO_2 tested with different methanol to water ratio, light source: 365 nm with the light intensity of 500 W/m^2, catalyst amount: 50 mg, temperature: 40℃; (**d**) AQE of CuSA-TiO_2 and Pt-TiO_2 tested under 365 nm light source with various light intensity, catalyst amount: 50 mg, methanol to water ratio: 2:1, temperature: 40℃.

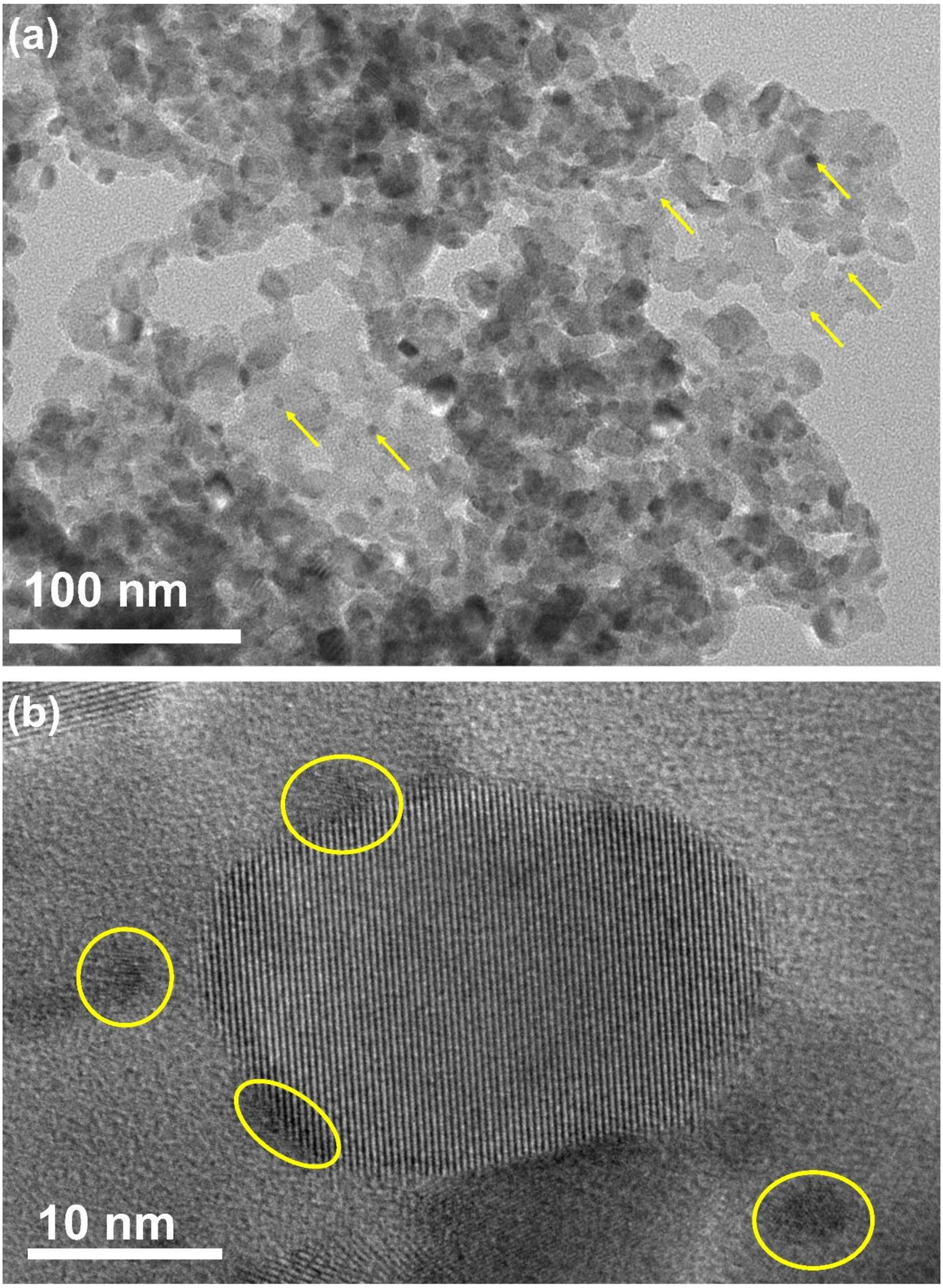

Supplementary Figure 2 | Morphology of the sample with higher Cu SAc loading. (**a**) TEM image and (**b**) HRTEM image of the sample with 2.57 wt% Cu-loading.

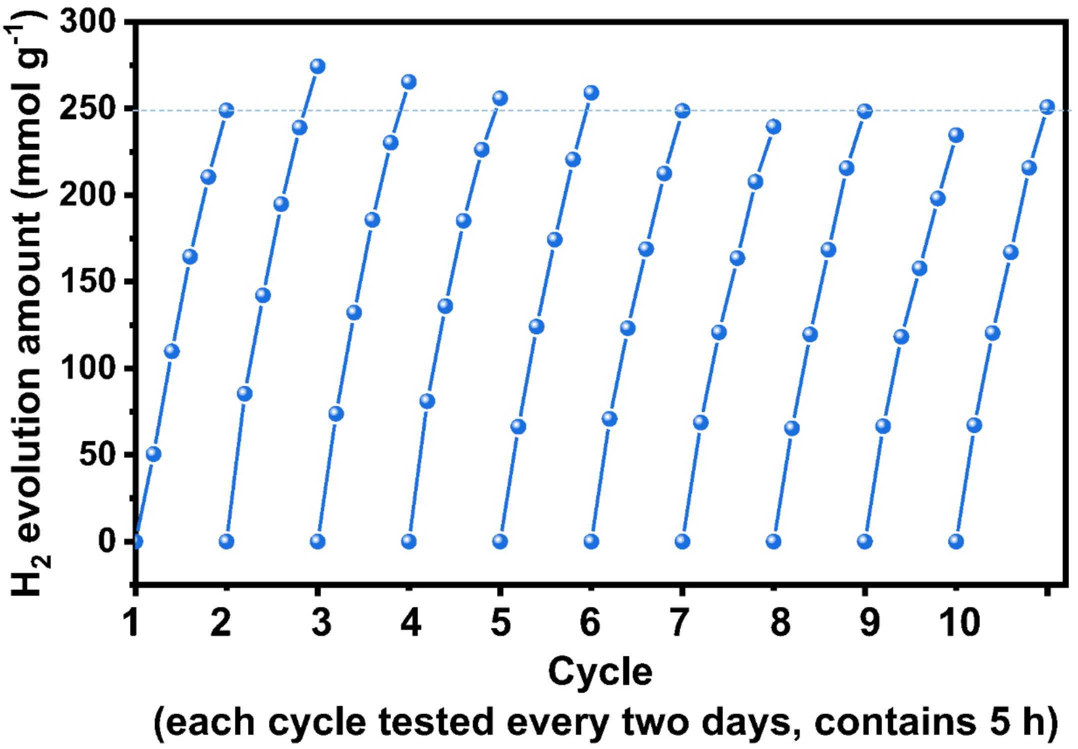

Supplementary Figure 3 | Long-term stability and reproducibility of CuSA-TiO_2 investigated by 10 cycles and 5 hours for one cycle (overall 20 days). In detail, 20 mg CuSA-TiO_2 was added to 120 mL solution of 2:1 methanol/water under 325 W/m^2 365 nm irradiation. The one-cycle activity was tested every two days without any condition change. To make up the evaporated solution and used methanol, 30 mL methanol was added in the run 5 and 30 mL 2:1 methanol/water in run 8. Until the last cycle, the sample has been kept in the solution for 20 days and it still exhibited similar activity to the fresh material. In this 10-cycle test, the hydrogen evolution activity is not as high as the cycling activity shown in **Fig. 1c in MS**. It is because of the light intensity attenuation of the Xe lamp from 500 W/m^2 to 325 W/m^2 for the safety issue for such a long time run. It is worth mentioning that the AQE remains similar under all these conditions, close to 56%.

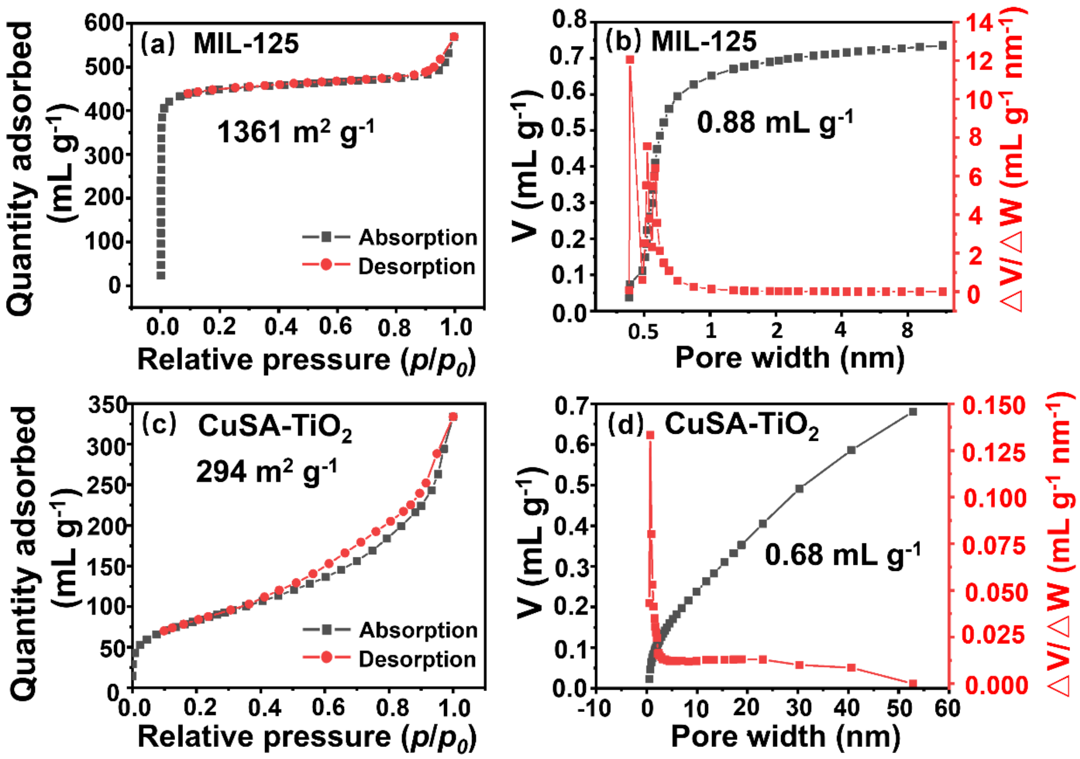

Supplementary Figure 4 | BET test of the MIL-125 precursor and CuSA-TiO_2. Nitrogen adsorption and desorption isotherms (**a, c**) and pore width distribution (**b, d**) of MIL-125 and CuSA-TiO_2.

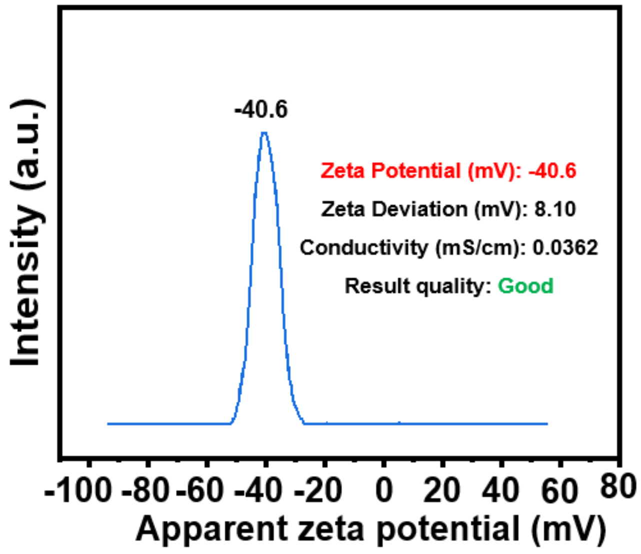

Supplementary Figure 5 | The result of the Zeta potential test of MIL-125(Ti_v). 1 mg of the samples were dispersed in 5 mL H_2O, and then underwent the test.

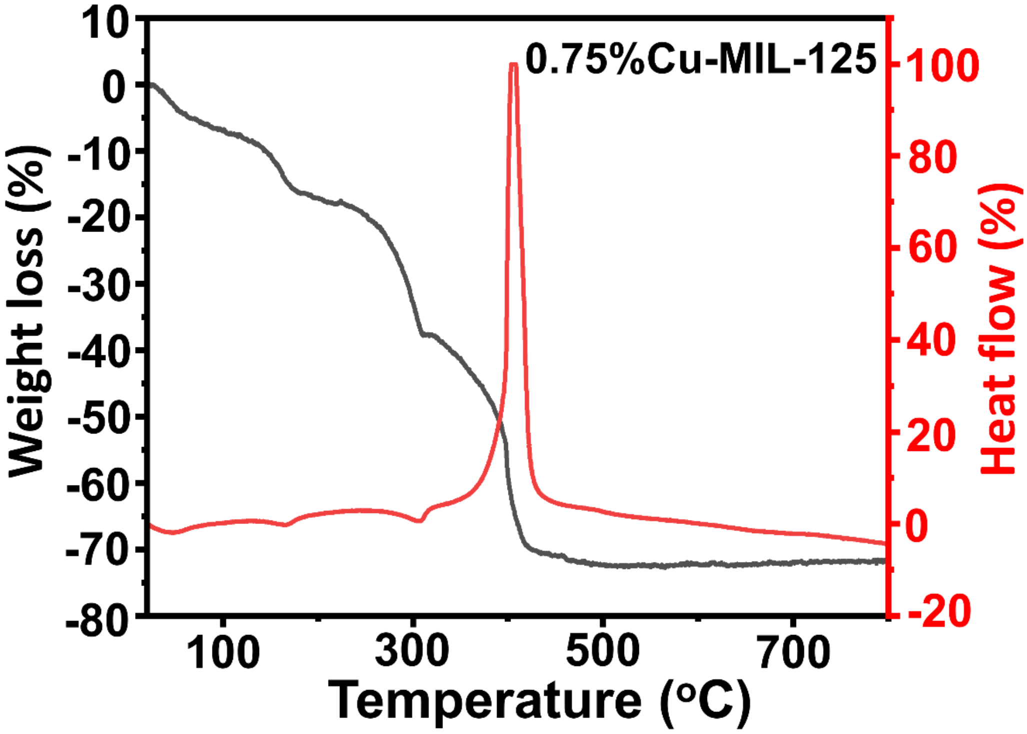

Supplementary Figure 6 | TG-DTA analysis. TG-DTA curve of 0.75% Cu-loaded MIL-125.

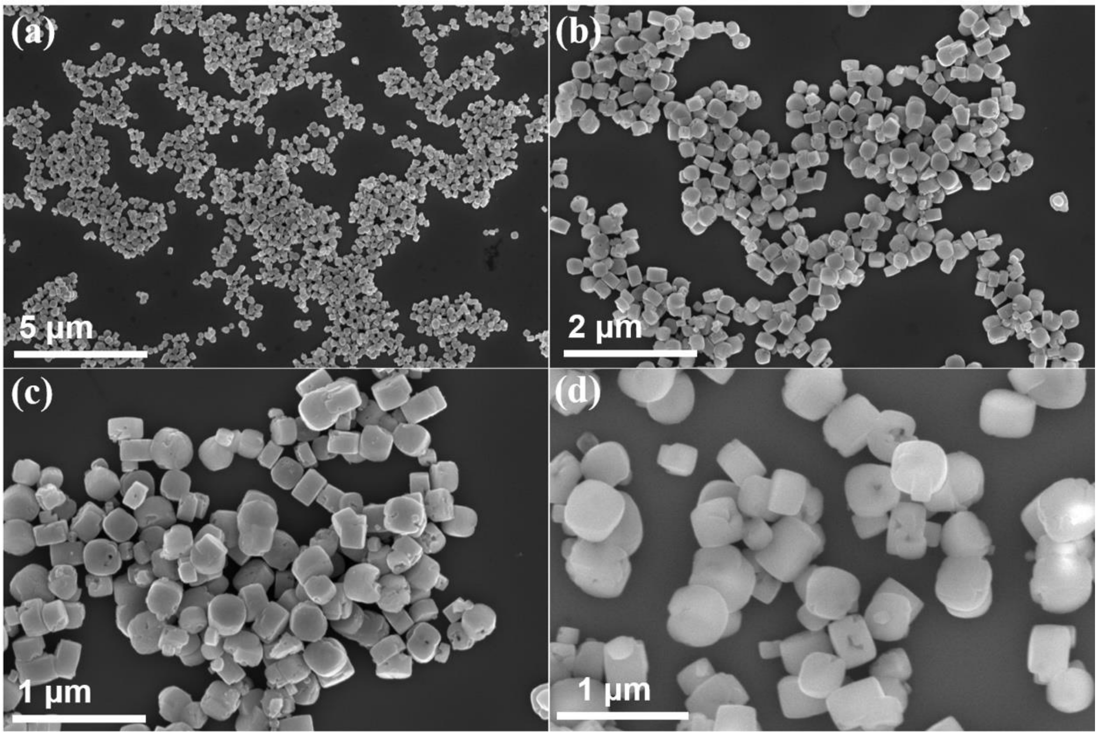

Supplementary Figure 7 | SEM images of MIL-125 with different magnifications. Low magnification SEM images of CuSA-TiO_2 with scale bars of (**a**) 5 µm, (**b**) 2 µm, (**c**) 1 µm, and (**d**) 1 µm.

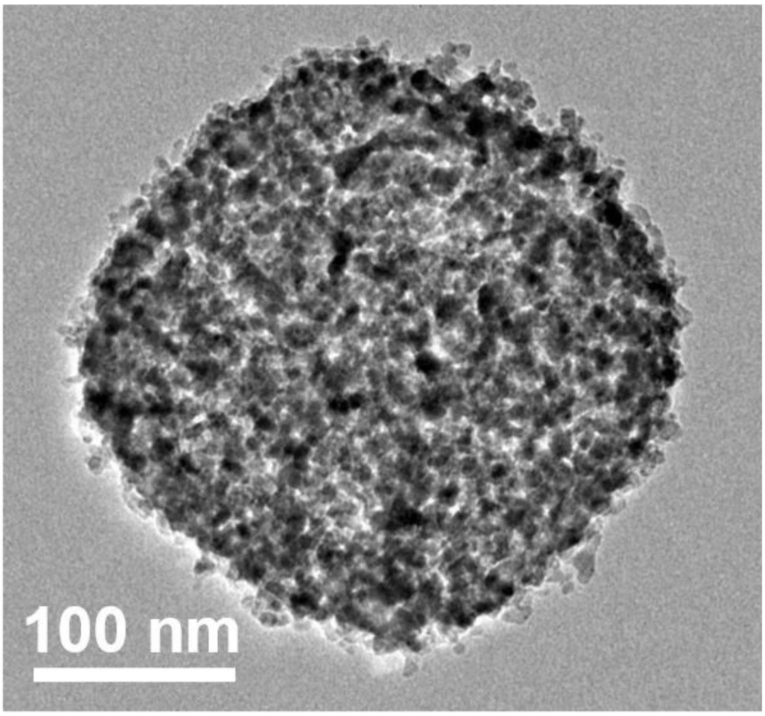

Supplementary Figure 8 | Morphology of pure TiO_2 derived from pure MIL-125. Low magnification TEM images of pure TiO_2 derived from pure MIL-125.

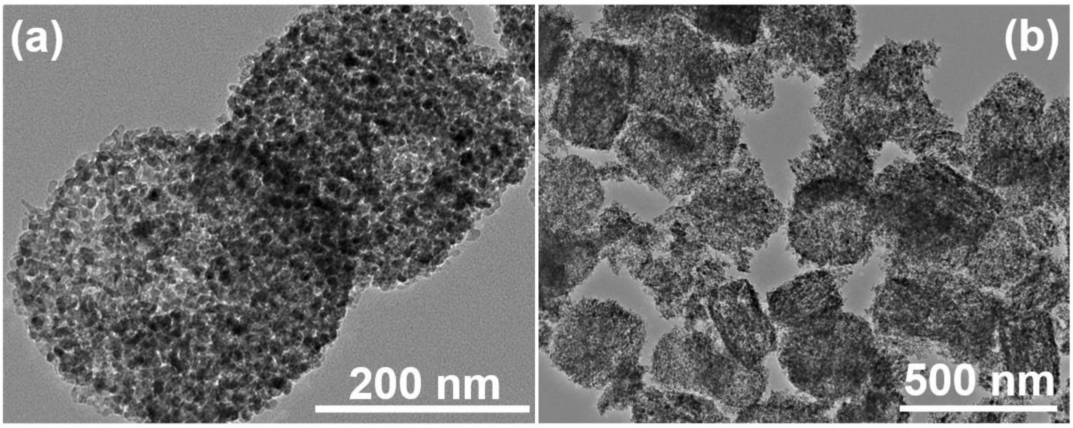

Supplementary Figure 9 | Low magnification TEM images of CuSA-TiO_2. Low magnification TEM images of CuSA-TiO_2 with scale bars of (**a**) 200 nm, and (**b**) 500 nm.

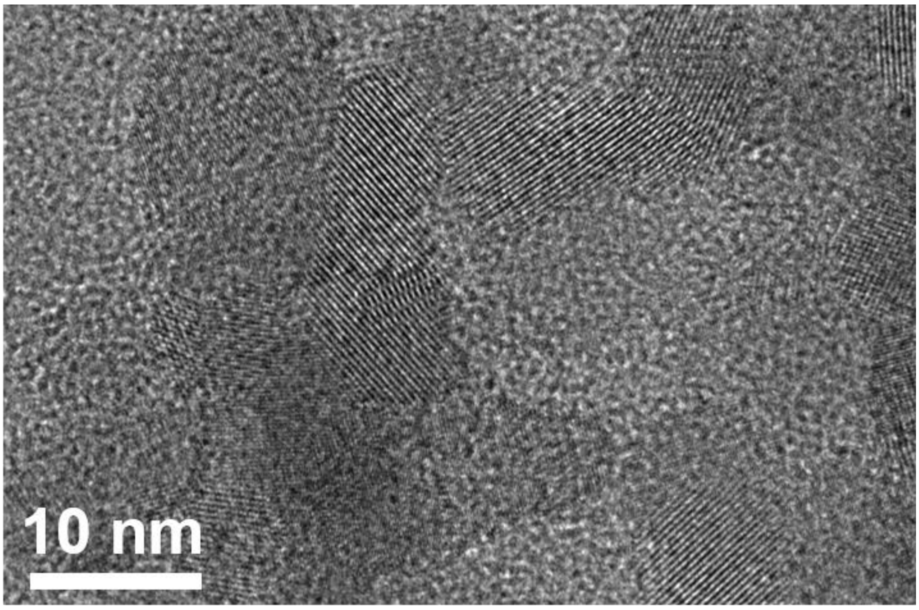

Supplementary Figure 10 | Micro morphology analysis. High magnification TEM images of CuSA-TiO_2.

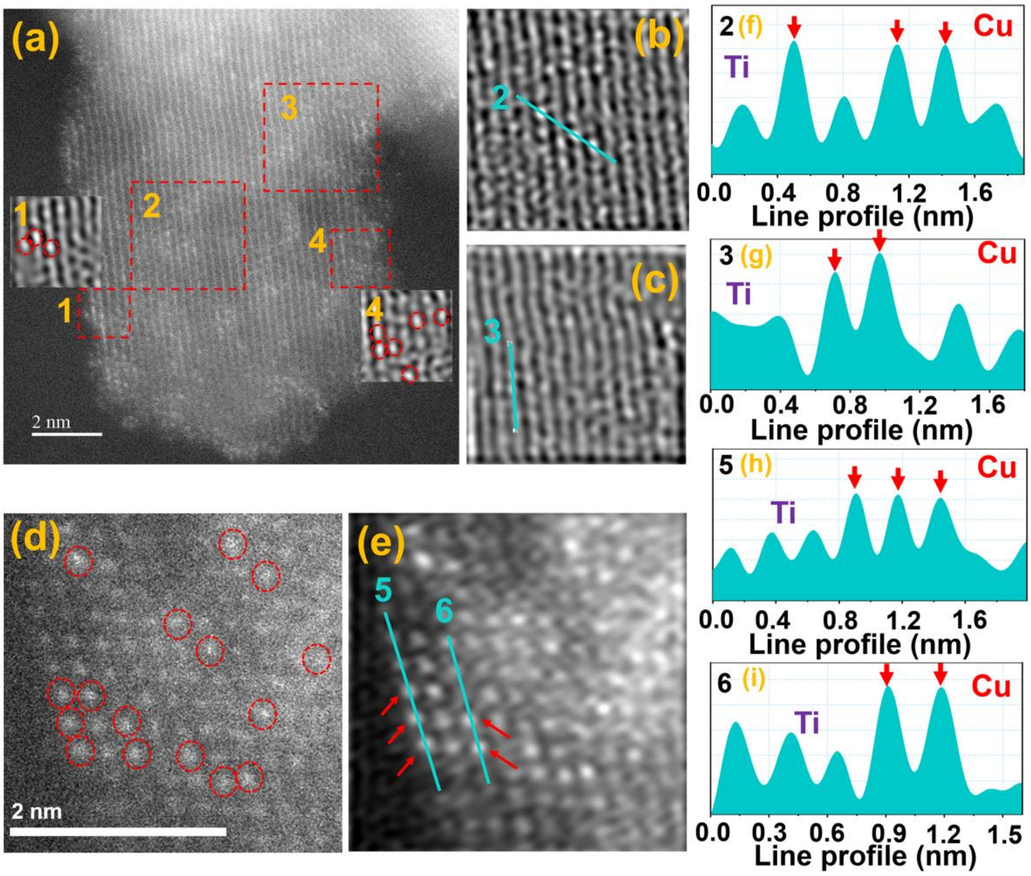

Supplementary Figure 11 | More HAADF STEM analysis of CuSA-TiO_2. Raw (**a, d**) and filtered image (**b, c, e**) as well as their line scan profile (**f-i**) of CuSA-TiO_2.

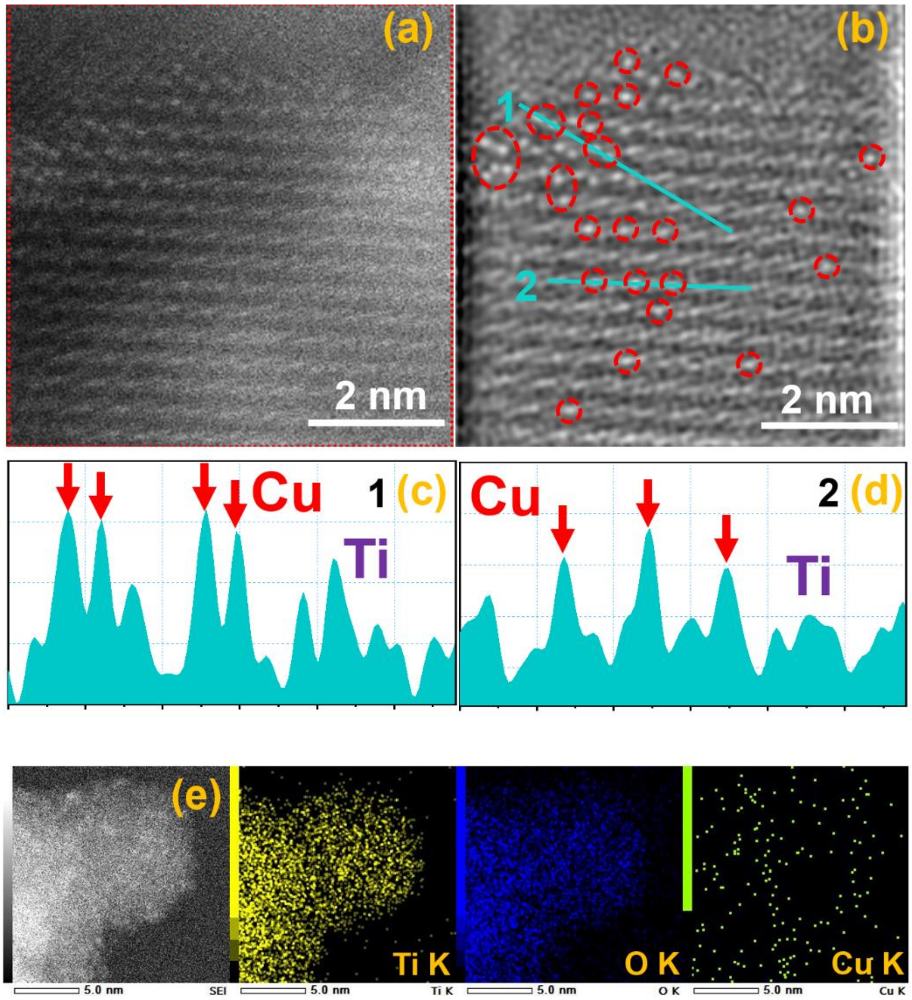

Supplementary Figure 12 | HAADF STEM analysis of CuSA-TiO_2 after 24-h photocatalytic reaction. (**a**) HAADF STEM raw image of CuSA-TiO_2 after 24-h photocatalytic reaction and its filtered HAADF STEM image (**b**), partially isolated Cu atoms are marked as dashed circles. (**c-d**) Line scan profile marked with blue lines 1 and 2 measured from (b). (**e**) STEM-EDS mapping of Ti, O, and Cu of CuSA-TiO_2 after 24-h photocatalytic reaction.

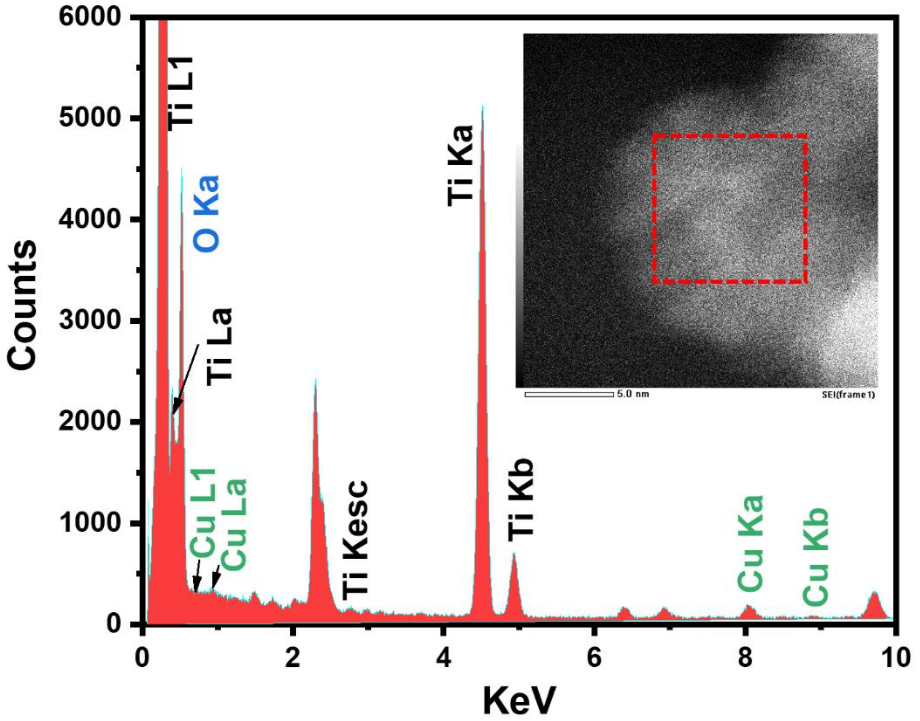

Supplementary Figure 13 | EDS analysis. EDS analysis of the selected area of CuSA-TiO_2.

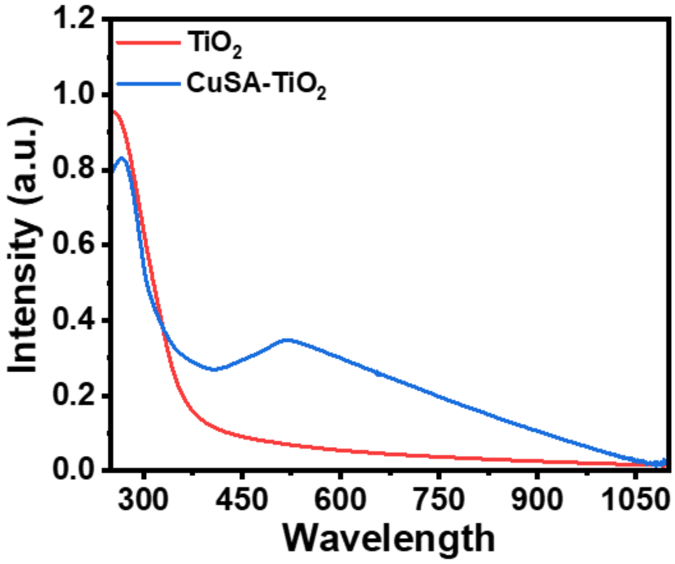

Supplementary Figure 14 | UV-vis absorption analysis. UV-vis absorption spectra of TiO_2 and CuSA-TiO_2.

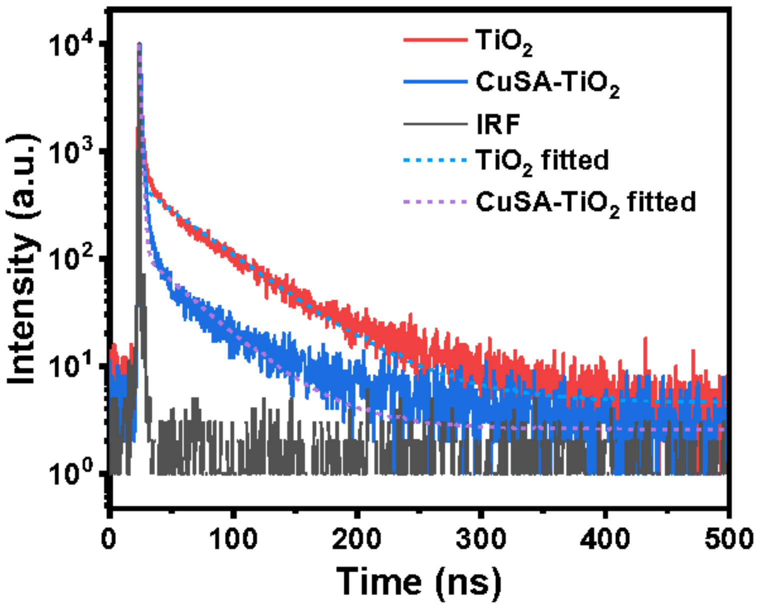

Supplementary Figure 15 | Fluorescence decay curves of TiO_2 and CuSA-TiO_2. The data was obtained by an FLS 1000 fluorescence spectrophotometer (UK) using 375 nm and 430 nm as excitation and detection wavelengths.

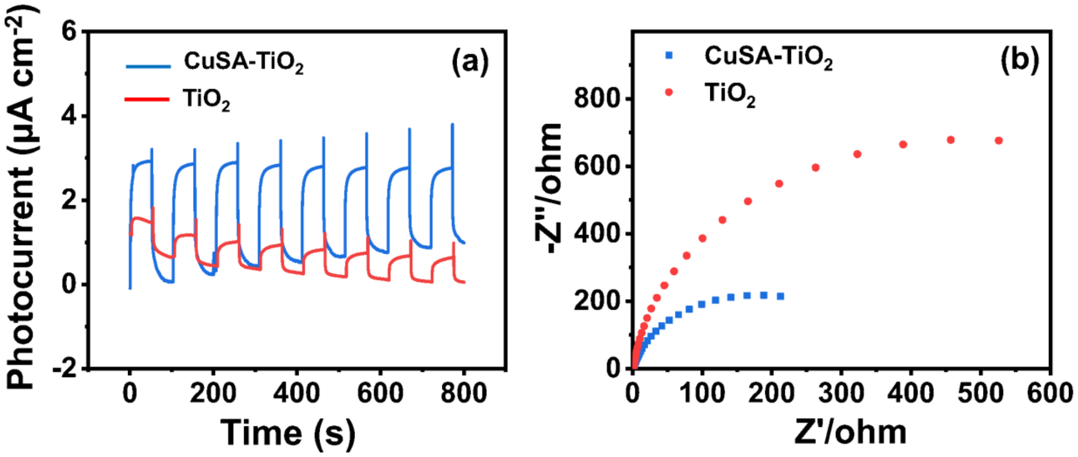

Supplementary Figure 16 | Photoelectric property analysis. (**a**) Photocurrent response of TiO_2 and 1.5 wt% CuSA-TiO_2. To do the test, the samples were compacted on foam Ni. It is the same for the EIS test. (**b**) EIS of TiO_2 and 1.5 wt% CuSA-TiO_2.

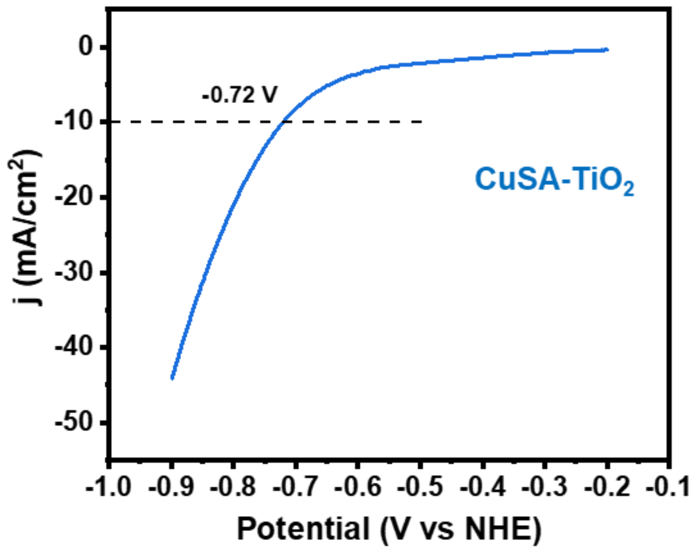

Supplementary Figure 17 | Electrochemical linear sweep voltammetry of CuSA-TiO$_2$. The prepared electrode, Pt wire, and Ag/AgCl electrode were used as the working, counter, and reference electrodes, respectively. The 0.2 M Na_2SO_4 aqueous solution was used as an electrolyte, and the photoelectrodes were irradiated using a 150 W xenon lamp.

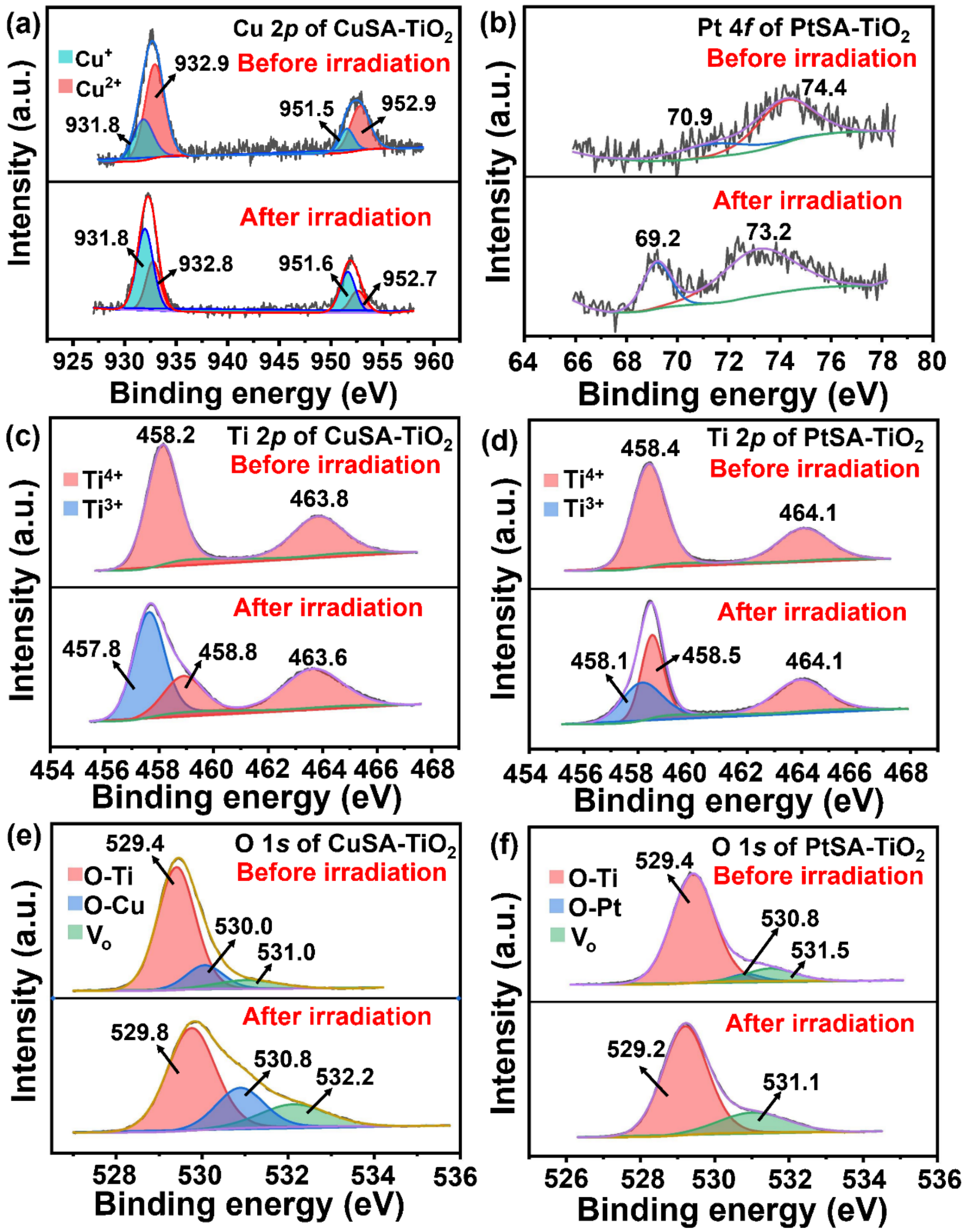

Supplementary Figure 18 | In-situ XPS results of Cu 2*p*, Pt 4*f*, Ti 2*p* and O 1*s*. In-situ Cu 2*p* (**a**), Pt 4*f* (**b**), Ti 2*p* (**c-d**) and O 1*s* (**e-f**) XPS of CuSA-TiO_2 and PtSA-TiO_2 before and after 30 min irradiation (LED 365 nm).

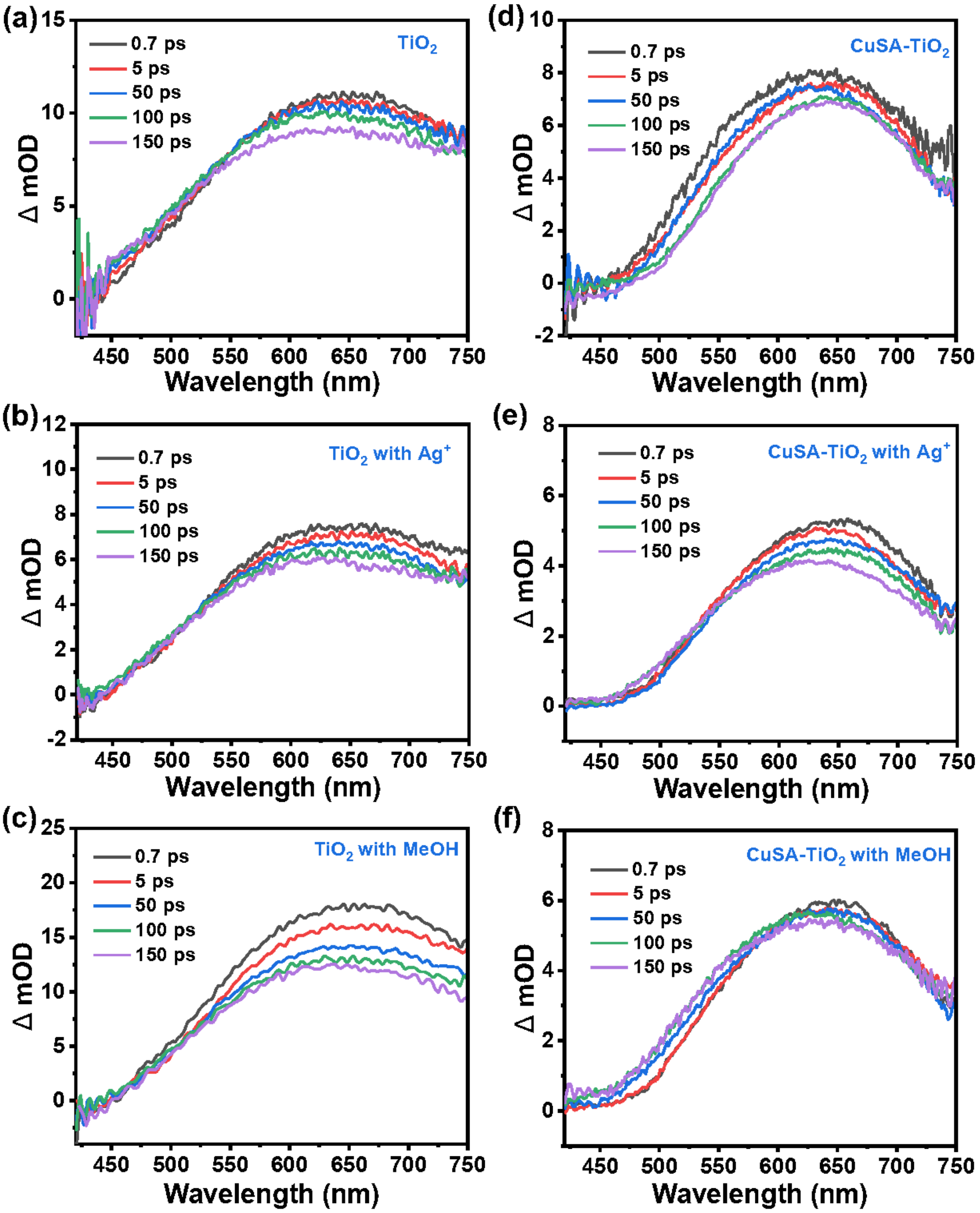

Supplementary Figure 19 | Transient absorption analysis. Transient absorption spectra of (**a**) TiO_2, (**b**) TiO_2 with Ag^+, (**c**) TiO_2 with MeOH, (**d**) CuSA-TiO_2, (**e**) CuSA-TiO_2 with Ag^+ and (**f**) CuSA-TiO_2 with MeOH.

One can note that the photoelectron signal (peaking at 650 nm) was slightly bigger in the absence of methanol than that in the presence of methanol on CuSA-TiO_2. We think this was due to the stronger photohole attraction of photoelectron in the former case than in the latter one, resulting into more photoelectrons on the surface of the photocatalyst in the former case while these photoelectrons were trapped by Cu SAs when photoholes were effectively scavenged by methanol.

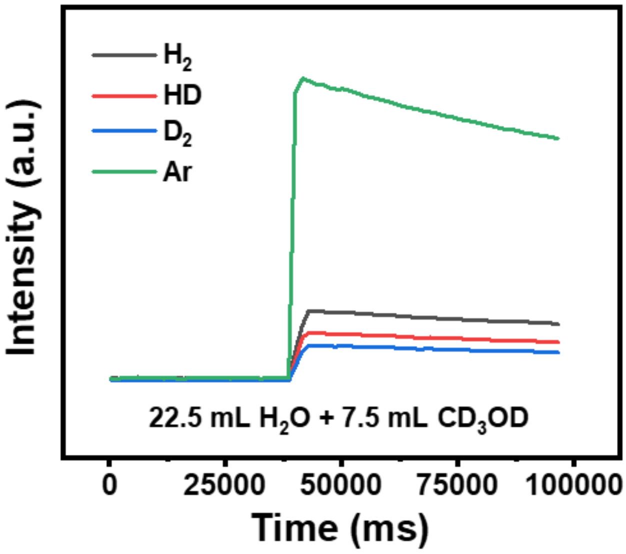

Supplementary Figure 20 | Isotopic analysis. Photocatalytic H_2, HD, and D_2 evolution on CuSA-TiO_2 from CD_3OD-H_2O case.

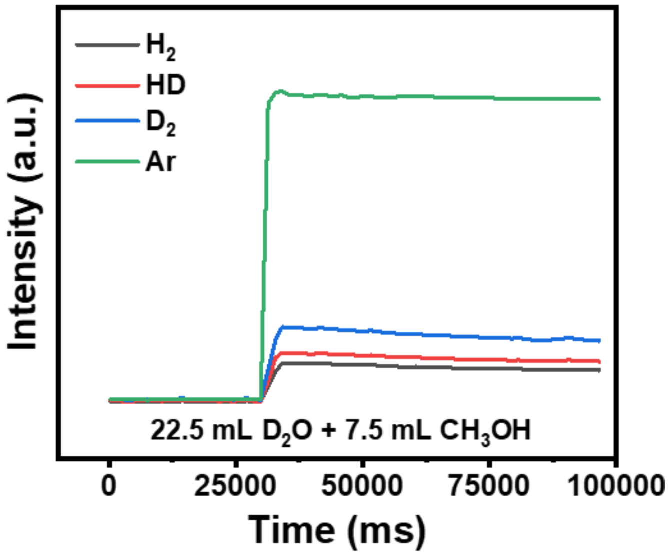

Supplementary Figure 21 | Isotopic analysis. Photocatalytic H_2, HD, and D_2 evolution on CuSA-TiO_2 from CH_3OH-D_2O case.

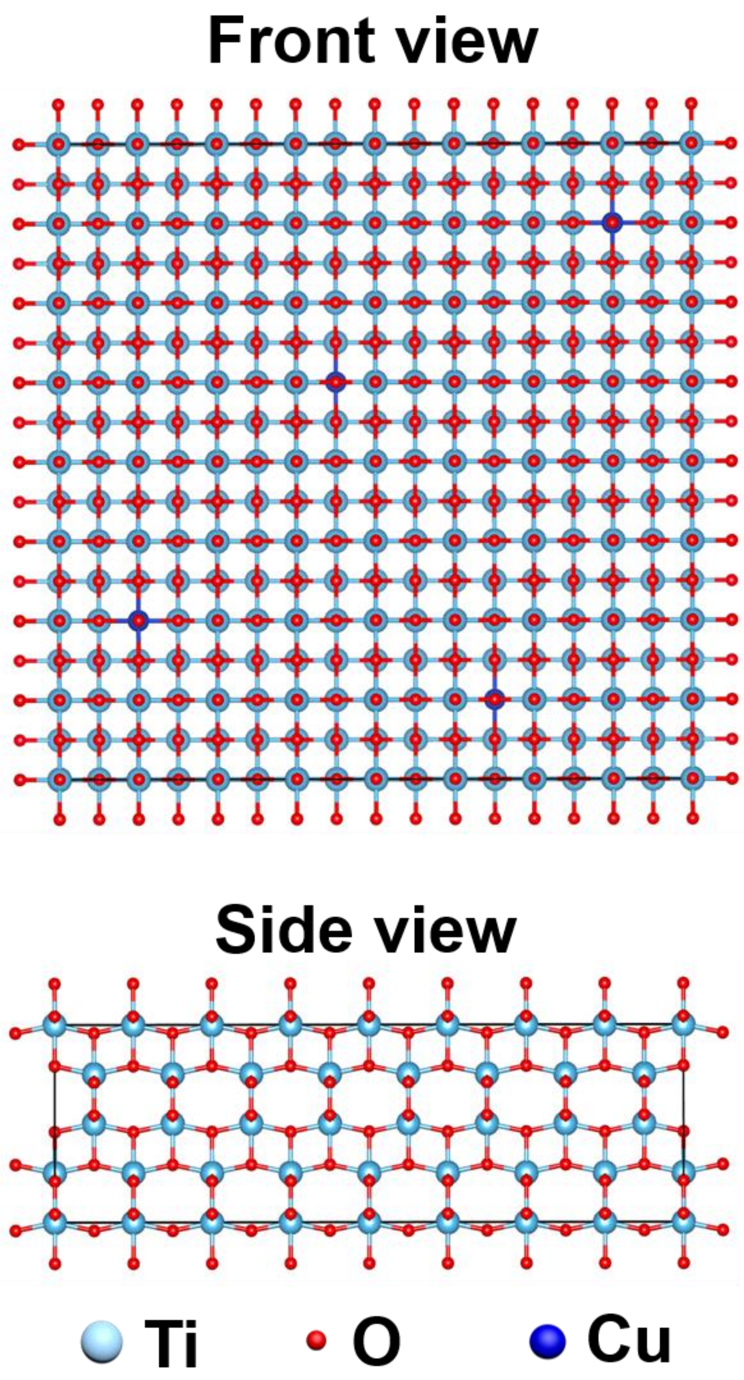

Supplementary Figure 22 | Theoretic simulations. The density functional theory simulations of 1.5wt% CuSA-TiO_2.

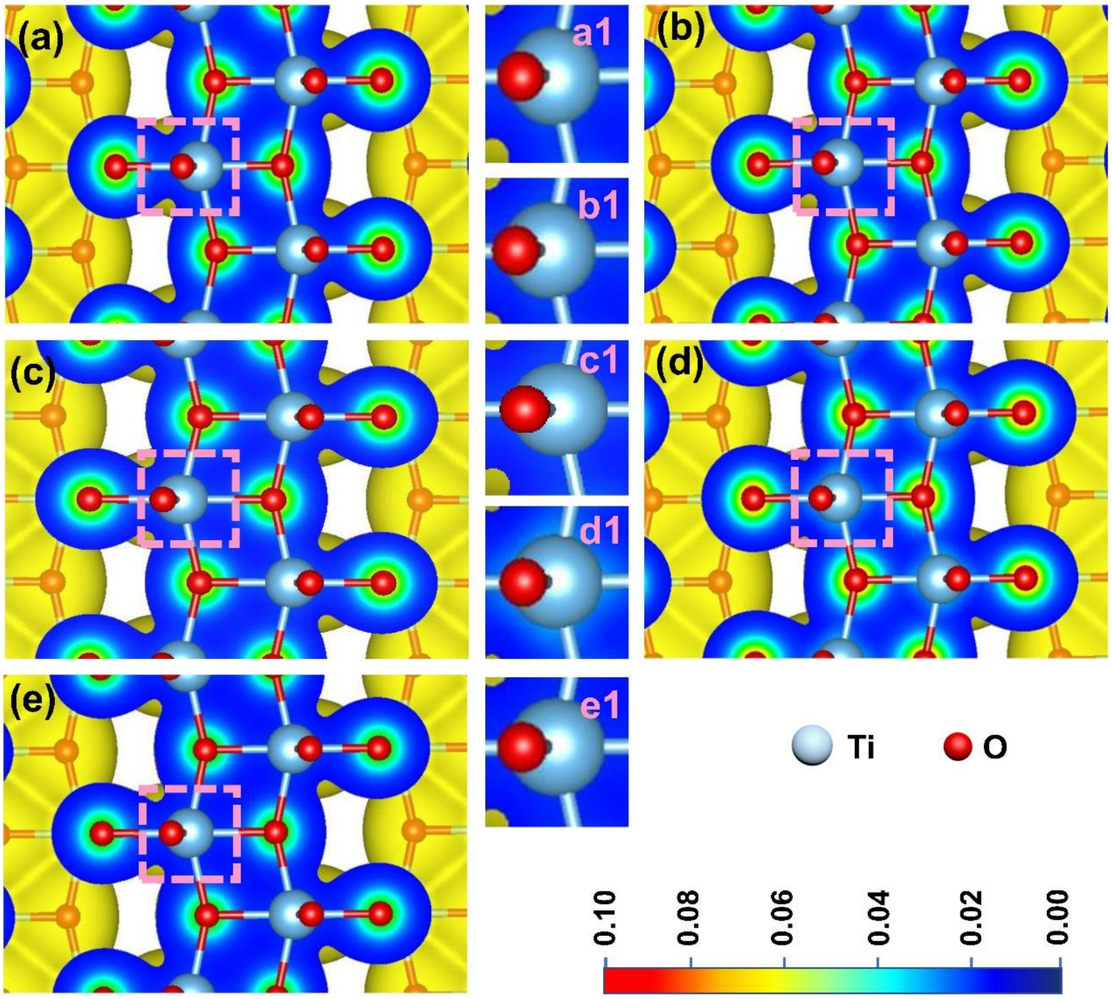

Supplementary Figure 23 | Charge density distribution of TiO_2 with or without irradiation. (**a**) Charge density distribution of pure TiO_2 under dark condition, (**b**) TiO_2 after 100 ps irradiation, (**c**) TiO_2 after 200 ps irradiation, (**d**) TiO_2 after 1 ns irradiation, (**e**) TiO_2 after 100 ps exposing in air, i.e. 100 ps after turning off the irradiation. Panel (a1-e1) is the magnification of the atom highlighted by pink square in (a-e).

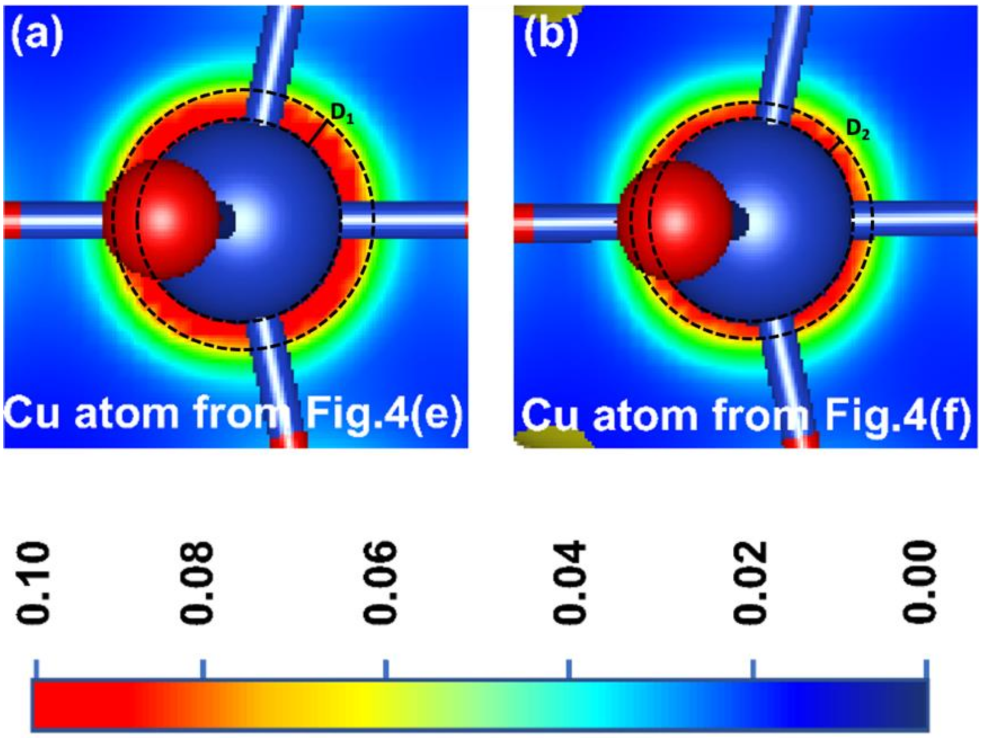

Supplementary Figure 24 | Partially magnified charge density distribution. Charge density on Cu (**a**) when irradiated for 1ns and (**b**) irradiation stops for 100 ps.

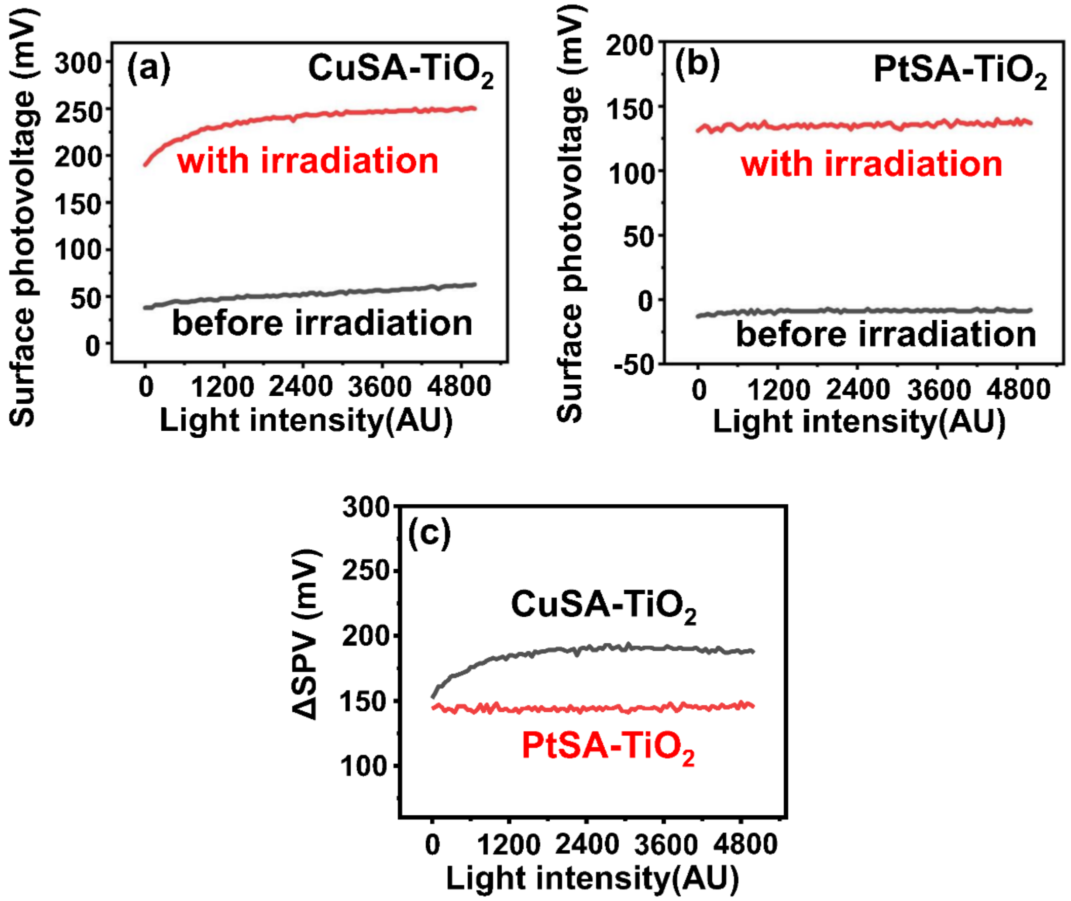

Supplementary Figure 25 | surface photovoltage analysis. The surface photovoltage of CuSA-TiO_2 (**a**) and PtSA-TiO_2 (**b**) before and after irradiation (LED 365 nm). (**c**) Comparison of the SPV between CuSA-TiO_2 and PtSA-TiO_2 before and after irradiation.

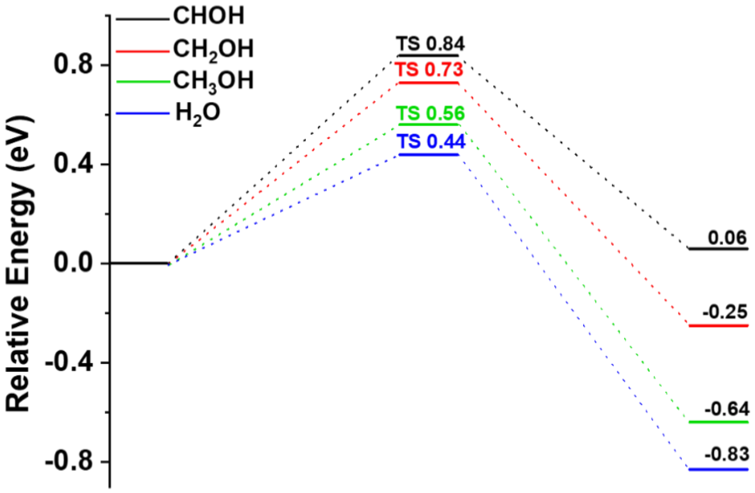

Supplementary Figure 26 | Potential energy. Potential energy diagram of photocatalytic H_2O reduction on Cu^+ and CH_3OH oxidation on Cu^{2+} ("TS" means transition state).

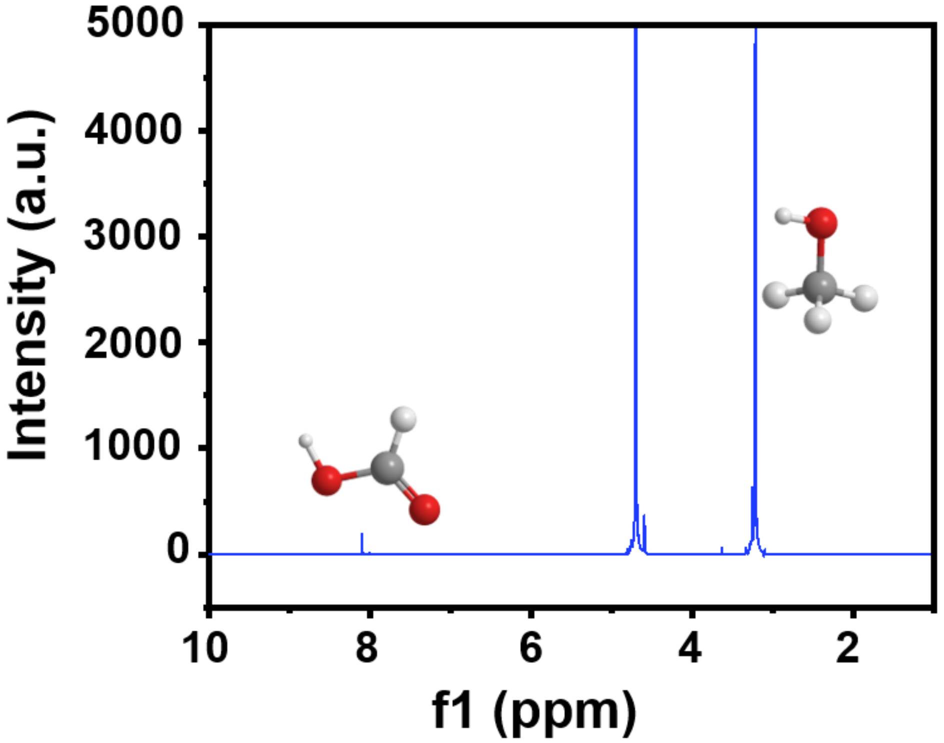

Supplementary Figure 27 | Component analysis of the reacted solution. Nuclear magnetic resonance spectrum of the solution from D_2O/CH_3OH solution after reaction.

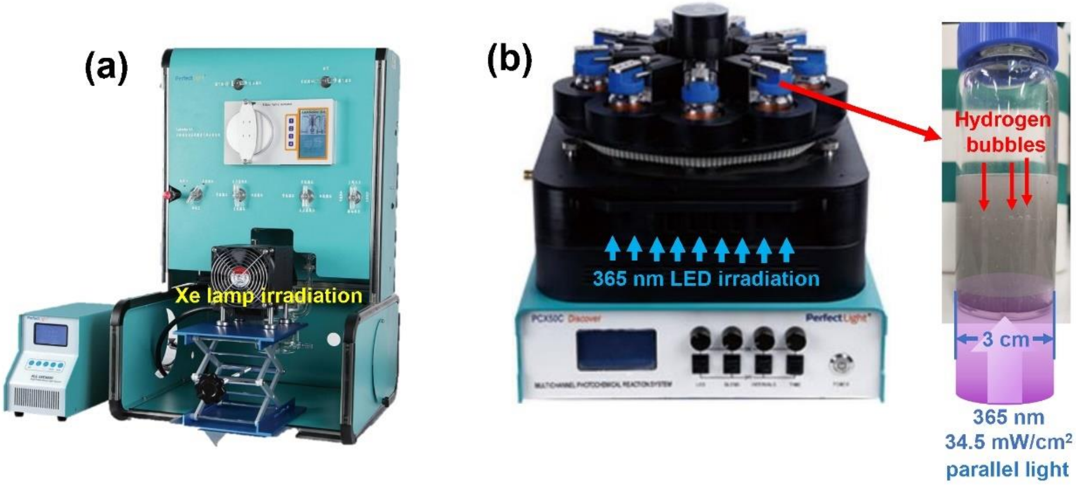

Supplementary Figure 28 | Photocatalytic H_2 evolution test equipment. (**a**) Full glass automatic on-line trace gas analysis system with Xe lamp (Labsolar-6A, Perfect Light Ltd.), which is used for the photocatalytic H_2 evolution test. (**b**) Multichannel photochemical reactor with 365 nm LED (PCX-50C, Perfect Light Ltd.). The H_2 evolution rate was measured by this equipment, which is used for AQE and isotopic test.

2. Supplementary Tables

Supplementary Table 1 | Parameters from inductively couple plasma spectroscopy of Metal-TiO_2

Sample	Metal ratio to MIL-125 precursor (wt.%)	Sample amount (mg)	Solvent amount (L)	Metal concentration (mg/L)	Metal ratio (wt.%)
1-Cu-TiO_2	0.25	112.4	0.05	10.803	0.48
2-Cu-TiO_2	0.50	112.5	0.05	23.415	1.04
3-Cu-TiO_2-1	0.75	112.2	0.05	33.1	1.48
3-Cu-TiO_2-2	0.75	115.6	0.05	34.63	1.50
3-Cu-TiO_2-3	0.75	112.8	0.05	33.3	1.48
3-Cu-TiO_2 after reaction	0.75	17.9	0.05	5.51	1.54
4-Cu-TiO_2	1.00	113.8	0.05	46.073	2.02
5-Cu-TiO_2	1.25	113.8	0.05	58.398	2.57
Co-TiO_2	0.75	113.3	0.05	38.747	1.71
Mn-TiO_2	0.75	115.8	0.05	22.351	0.97
Zn-TiO_2	0.75	113.1	0.05	34.827	1.54
Fe-TiO_2	0.75	35.6	0.05	6.7197	0.94
Ni-TiO_2	0.75	95.6	0.05	35.753	1.87
Pt-TiO_2	0.75	76.8	0.05	9.7555	0.64

**Supplementary Table 2 丨 Comparison of photocatalytic activity in PHE on recent TiO_2-based or MOF-based photocatalyst**						
**Catalysts**	**Specific surface area (m^2g^{-1})**	**Light source**	**Hydrogen evolution rate (mmol g^{-1} h^{-1})**	**Apparent quantum efficiency**	**Stability**	**Ref.**
**TiO_2 based photocatalysts**						
**5%CNT-La-TiO_2**	135	Xe lamp (35W)	8.86	--	--	2
**Ni cluster -TiO_2**	--	Xe lamp (350W)	8.72	--	Storage for 100 days	3
**0.6%Pt atom-TiO_2**	--	Xe lamp (300W)	21.90	--	--	4
**0.5%Pt atom-TiO_2**	--	Xe lamp (300W)	9.00	--	--	5
**0.46%Ni atom-TiO_2**	--	Xe lamp (300W)	1.90	--	Reaction for 4 cycles (20 h)	6
**0.5%Pt atom-TiO_2**	--		1.80	--	--	
**Sb-N-TiO_2**	55.8	Xe lamp (300W)	2.33	--	--	7
**0.75%Cu atom-TiO_2**	--	340 nm Xe lamp	16.60	45.5%	Reaction for 4 cycles (20 h)	8
**0.99%Pt atom-TiO_2**	--	Xe lamp (300W)	52.72	--	Reaction for 5 cycles (10 h)	9
**MOF based photocatalysts**						
**0.36%Ru@N-TiO_2/C derived from MOF**	307.5	Xe lamp (300W)	5.00	--	Reaction for 3 cycles (9 h)	10
**0.1%Pt@MOF-Al-TCPP**		Xe lamp (300W)	0.13	--	Reaction for 4 cycles (4 h)	11
**1.52%Pt@ MOF-MNS**	570	>420 nm Xe lamp	11.32	--	Reaction for 4 cycles (4 h)	12
**CuSA-TiO_2**	294	Xe lamp (500W)	101.74	56% (365nm)	Storage for 380 days and reaction for 6 cycles (30 h)	This work
**PtSA-TiO_2**	210	Xe lamp (500W)	95.26	--		

**Supplementary Table 3 丨 The fitted PL decay results of TiO_2 and CuSA-TiO_2**					
**Sample**	**τ_1 (ns)**	**τ_2 (ns)**	**B_1**	**B_2**	**τ_{ave} (ns)[a]**
**TiO_2**	1.16	50.83	1.018839	0.057672	3.82
**CuSA-TiO_2**	1.24	40.68	1.004658	0.020758	2.04

[a] **τ_{ave} is calculated by $(B_1\tau_1 + B_2\tau_2) / (B_1+B_2)$**

Supplementary Table 4 | The XPS peak area and atomic percentage of Cu in CuSA-TiO_2 before and after photocatalytic reactions

Catalyst	Catalyst state	Peak area	Atomic (%)	Peak area	Atomic (%)
		Cu^{2+}		Cu^{+}	
CuSA-TiO_2	Before irradiation	8137+16786	70.58	7534+2854	29.42
	Irradiation 30 min	7191+5989	38.32	4718+16498	61.68
	Irradiation 60 min	8669+4278	33.63	17134+8427	66.37
	Exposing in air	12279+5284	46.46	13991+6249	53.54

Supplementary Table 5 | The XPS peak area and atomic percentage of the different elements in CuSA-TiO_2

	Elements	Before irradiation		After irradiation	
		Peak area	Atomic (%)	Peak area	Atomic (%)
CuSA-TiO_2	**Ti 2p**				
	Ti^{4+}	126548+65253	100	63314+39116	52.98
	Ti^{3+}	0	0	90873	47.02
	**O 1s**				
	O-Ti	171497	76.66	151628	58.90
	O-Cu	32504	14.53	58948	22.89
	Vo	19723	8.81	46877	18.21

Supplementary Table 6 | The XPS peak area and atomic percentage of the different elements in PtSA-TiO_2

Catalyst	Elements	Before irradiation		After irradiation	
		Peak area	Atomic (%)	Peak area	Atomic (%)
PtSA-TiO_2		**Ti 2p**			
	Ti^{4+}	188008+89770	100	290031+58858	86.88
	Ti^{3+}	0	0	52681	13.12
		**O 1s**			
	O-Ti	117377	84.70	234958	78.00
	O-Pt	5655	4.10		
	Vo	15543	11.20	66282	22.00

Supplementary Table 7 丨 The XPS peak area and atomic percentage of the different elements in CuSA-TiO_2				
**Catalyst state**	**EPR peak area**	**Inferred Cu^{2+} proportion from EPR**	**XPS Cu^{2+} proportion originated in Table S3**	**Inferred Cu^{2+} proportion from XPS**
Before irradiation	6221.1	100%	70.58%	100%
Irradiation 30 min	3561.8	57.25%	38.32%	54.29%
Irradiation 60 min	1311.1	21.08%	33.63%	47.64%
Exposing in air	4142.2	66.58%	46.46%	65.82%

The Cu^{2+} contents estimated from EPR data are almost consistent with the XPS data and the comparison of measuring Cu^{2+} under the different exposing conditions is given in Table S7. The peak area observed before irradiation is defined as 100% Cu^{2+} as the Cu^{+} signal is silent. The Cu^{2+} proportions corresponding to the other conditions were calculated and are shown in Table S7. Also, the Cu^{2+} proportion obtained from XPS was given in the same table in the green column. The Cu^{2+} estimated before irradiation was regarded as 100% and the percentages of Cu^{2+} for the other conditions are estimated by taking this as a reference (the blue column). The Cu^{2+} proportion measured by EPR and XPS is quite similar except for "irradiation 60 min". The lower Cu^{2+} content estimated in EPR under this condition may be because the light intensity during EPR measurement was much stronger than the one used in XPS, hence the conversion of Cu^{2+} to Cu^{+} is faster during the second 30 min irradiation in EPR. Furthermore, the XPS results are related to the surface Cu^{2+} while EPR has information from the bulk so the former is more useful for analysis of the catalytic activity.

References:

1. Dan-Hardi, M. et al. A new photoactive crystalline highly porous titanium(IV) dicarboxylate. *J. Am. Chem. Soc.* **131**, 10857-10859 (2009).
2. Tahir, M. La-modified TiO_2/carbon nanotubes assembly nanocomposite for efficient photocatalytic hydrogen evolution from glycerol-water mixture. *Int. J. Hydrogen Energy* **44**, 3711-3725 (2019).
3. Si, J. *et al.* Colour centre controlled formation of stable sub-nanometer transition metal clusters on TiO_2 nanosheet for high efficient H_2 production. *Appl. Surf. Sci.* **511**, 145577 (2020).
4. Wei, T., Zhu, Y., Wu, Y., An, X. & Liu, L.-M. Effect of Single-Atom Cocatalysts on the Activity of Faceted TiO_2 Photocatalysts. *Langmuir* **35**, 391-397 (2019).
5. Jeantelot, G. et al. TiO_2-supported Pt single atoms by surface organometallic chemistry for photocatalytic hydrogen evolution. *PCCP* **21**, 24429-24440 (2019).
6. Xiao, M. et al. Molten-Salt-Mediated Synthesis of an Atomic Nickel Co-catalyst on TiO_2 for Improved Photocatalytic H-2 Evolution. *Angewandte Chemie-International Edition* **59**, 7230-7234 (2020).
7. Lv, Z. et al. Enhanced photoredox water splitting of Sb-N donor-acceptor pairs in TiO_2. *Inorg. Chem. Front.* **6**, 2404-2411 (2019).
8. Lee, B. H. et al. Reversible and cooperative photoactivation of single-atom Cu/TiO_2 photocatalysts. *Nat. Mater.* **18**, 620-626 (2019).
9. Chen, Y. et al. Engineering the Atomic Interface with Single Platinum Atoms for Enhanced Photocatalytic Hydrogen Production. *Angew Chem Int Ed Engl* **59**, 1295-1301 (2020).
10. Yan, B., Liu, D., Feng, X., Shao, M. & Zhang, Y. Ru Species Supported on MOF-Derived N-Doped TiO_2/C Hybrids as Efficient Electrocatalytic/Photocatalytic Hydrogen Evolution Reaction Catalysts. *Adv. Funct. Mater.*, 2003007 (2020).
11. Fang, X. et al. Single Pt Atoms Confined into a Metal-Organic Framework for

Efficient Photocatalysis. *Adv. Mater.* **30**, 1705112 (2018).

12. Zuo, Q. et al. Ultrathin Metal-Organic Framework Nanosheets with Ultrahigh Loading of Single Pt Atoms for Efficient Visible-Light-Driven Photocatalytic H_2 Evolution. *Angewandte Chemie-International Edition* **58**, 10198-10203 (2019).
